# A novel hypomorphic allele of *Spag17* causes primary ciliary dyskinesia phenotypes in mice

**DOI:** 10.1242/dmm.045344

**Published:** 2020-10-30

**Authors:** Zakia Abdelhamed, Marshall Lukacs, Sandra Cindric, Heymut Omran, Rolf W. Stottmann

**Affiliations:** 1 Division of Human Genetics, Cincinnati Children's Hospital Medical Center, Cincinnati, OH 45229, USA; 2 Department of Anatomy and Embryology, Faculty of Medicine (Girl's Section), Al-Azhar University, Cairo 11651, Egypt; 3 Medical Scientist Training Program, Cincinnati Children's Hospital Medical Center, Cincinnati, OH 45229, USA; 4 Department of General Pediatrics, University Children's Hospital Münster, 48149 Münster, Germany; 5 Division of Developmental Biology, Cincinnati Children's Hospital Medical Center, Cincinnati, OH 45229, USA; 6 Department of Pediatrics, University of Cincinnati, Cincinnati, OH 45229, USA

**Keywords:** Cilia, Hydrocephalus, Infertility, Lung, Primary ciliary dyskinesia, *Spag17*

## Abstract

Primary ciliary dyskinesia (PCD) is a human condition of dysfunctional motile cilia characterized by recurrent lung infection, infertility, organ laterality defects and partially penetrant hydrocephalus. We recovered a mouse mutant from a forward genetic screen that developed many of the hallmark phenotypes of PCD. Whole-exome sequencing identified this primary ciliary dyskinesia only (*Pcdo*) allele to be a nonsense mutation (c.5236A>T) in the *Spag17* coding sequence creating a premature stop codon (K1746*). The *Pcdo* variant abolished several isoforms of SPAG17 in the *Pcdo* mutant testis but not in the brain. Our data indicate differential requirements for SPAG17 in different types of motile cilia. SPAG17 is essential for proper development of the sperm flagellum and is required for either development or stability of the C1 microtubule structure within the central pair apparatus of the respiratory motile cilia, but not the brain ependymal cilia. We identified changes in ependymal ciliary beating frequency, but these did not appear to alter lateral ventricle cerebrospinal fluid flow. Aqueductal stenosis resulted in significantly slower and abnormally directed cerebrospinal fluid flow, and we suggest that this is the root cause of the hydrocephalus. The *Spag17^Pcdo^* homozygous mutant mice are generally viable to adulthood but have a significantly shortened lifespan, with chronic morbidity. Our data indicate that the c.5236A>T *Pcdo* variant is a hypomorphic allele of *Spag17* that causes phenotypes related to motile, but not primary, cilia. *Spag17^Pcdo^* is a useful new model for elucidating the molecular mechanisms underlying central pair PCD pathogenesis in the mouse.

This article has an associated First Person interview with the first author of the paper.

## Abstract (precis)

**Summary:** The *Spag17^pcdo^* mouse model closely recapitulates the human central pair primary ciliary dyskinesia condition, and the data here reinforce the power of the hypomorphic allele in representing human conditions.

## INTRODUCTION

Cilia are centriole-derived, microtubule-based membranous extensions that exist in almost every cell (Gilula and Satir, 1972; Pedersen et al., 2008; Satir, 2005). Ciliary structures are highly conserved across the animal kingdom and can be classified broadly into two main types, largely based on the structure of the ciliary axoneme. The nonmotile primary cilia have a ‘9+0’ axonemal structure (Nogales et al., 1999; Satir, 2005) because they lack the central pair of microtubules and other molecular motors, such as dynein arms and radial spokes, responsible for ciliary movement. These primary cilia are mainly involved in mechanosensory functions (Goetz and Anderson, 2010; Pazour et al., 2000; Sánchez and Dynlacht, 2016). Nodal cilia are motile cilia that lack the central pair apparatus but do have inner and outer dynein arms and can cause a leftward fluid flow to convey an asymmetric signaling event, resulting in asymmetric expression of laterality genes, such as *Pitx2* (Brueckner, 2001; Marszalek et al., 1999; Nonaka et al., 1998). The motile cilia develop a central pair of microtubule singlets in the center of the axoneme and are therefore described as having a ‘9+2’ arrangement of microtubule doublets (Nogales et al., 1999; Satir, 2005). The well-documented function of motile cilia is the coordinated rhythmic beating to move body fluids in the brain, respiratory tract and male and female genital ducts (Brightman and Palay, 1963; Dirksen, 1971; Jeffery and Reid, 1975). This is consistent with the localized tissue distribution of motile cilia to these organs.

Defects in the assembly or function of motile cilia can cause primary ciliary dyskinesia (PCD). PCD (OMIM: 244400) is a rare and highly heterogeneous condition, with variants in more than 40 causative genes reported to date (Bustamante-Marin et al., 2019; Cindrić et al., 2020; Lucas et al., 2019). In spite of this progress, a genetic diagnosis remains elusive in approximately 35% of PCD patients (Kurkowiak et al., 2015; Yang et al., 2018). PCD manifests as chronic respiratory tract infections, infertility, and laterality defects in around 50% of cases (Afzelius, 1981; Leigh et al., 2019). These laterality defects in PCD are thought to develop owing to defective nodal cilia in the developing embryo. Hydrocephalus occurs infrequently in individuals with PCD and might reflect dysfunctional ependymal cilia, although various other mechanisms have also been proposed (Berlucchi et al., 2012; Del Bigio, 2010; Kosaki et al., 2004; Lee, 2013; Vieira et al., 2012; Wessels et al., 2003). Intriguingly, most rodent models of PCD genes develop hydrocephalus, but there is significant evidence for phenotypic variability based on the genetic background (Abdelhamed et al., 2018; Brody et al., 2000; Fernandez-Gonzalez et al., 2009; Finn et al., 2014; Ha et al., 2016; Ibañez-Tallon et al., 2004; Lechtreck et al., 2008; Lee et al., 2008; Sironen et al., 2011).

Ciliary motility is an ATP-dependent process that results in activation of dynein motor proteins between the pairs of microtubule doublets that are the major structural component of the ciliary axoneme (Satir and Sleigh, 1990). A number of studies indicate that signals from the central pair propagate through radial spokes to modulate the dynein activity and, ultimately, affect ciliary beating and flagellar movement (Adams et al., 1981; Smith and Yang, 2004; Wirschell et al., 2009; Yang and Smith, 2009; Zhu et al., 2019). These studies generally conclude that dynein is a downstream effector of the central pair apparatus (Huang et al., 1982; Porter et al., 1994, 1992; Rupp et al., 1996). The central pair consists of the two 13-protofilament microtubule singlets (C1 and C2) interconnected by a bridge-like structure, in addition to several projections docked onto the C1 (C1a-C1f) and C2 (C2a-C2b) singlets. At least 23 polypeptides, ranging in molecular weight from 14 to 360 kDa, make up the central apparatus (Adams et al., 1981; Zhao et al., 2019). The C1 and C2 microtubule singlets and their associated projections are structurally and biochemically distinct. At least 10 different polypeptides are uniquely associated with the C1 microtubule, and seven are unique to the C2 microtubule (Dutcher et al., 1984; Lee, 2011). This biochemical and structural asymmetry is believed to have functional consequences for ciliary or flagellar beat and waveform (Inaba, 2011). Studies in *Chlamydomonas reinhardtii* indicate that the 240 kDa protein PF6 is the largest protein in the C1a projection (Goduti and Smith, 2012; Rupp et al., 2001), with multiple roles, such as acting as a scaffold protein essential for assembly of the smaller C1a components (Rupp et al., 2001), interaction with the calmodulin calcium-binding protein (Wargo et al., 2005) and, possibly, playing a role in modulating the activity of both inner and outer dynein arms (Goduti and Smith, 2012). As expected, *Chlamydomonas pf6* mutants develop paralyzed flagella and lack the central pair C1a projection (Rupp et al., 2001).

Sperm associated antigen 17 (*Spag17*) is the mammalian homolog of *Chlamydomonas pf6* (Zhang et al., 2005). SPAG17 protein is present in motile cilia and flagella with a ‘9+2’ axoneme structure (Zhang et al., 2005, 2007), including the ciliated ependymal cells (Gokce et al., 2016). Similar to its ortholog, the mammalian SPAG17 protein is present in the central pair apparatus of motile cilia and is essential for development of the C1a projection of the C1 microtubule singlet (Rupp et al., 2001; Teves et al., 2013; Zhang et al., 2007). SPAG17 and other C1 proteins, including SPAG6 and SPAG16L, are known to bind within the C1a projection (Zhang et al., 2005, 2007). Consistent with this biology, *Spag17* null mice develop severe ciliary motility defects that lead to hydrocephalus, severe respiratory distress and death within 12 h after birth (Teves et al., 2013). *Spag17* mutant mice also have phenotypes beyond the motile cilia, including skeletal defects, with shorter and fewer primary cilia on the *Spag17* mutant chondrocytes, osteoblasts and embryonic fibroblasts (MEFs) (Teves et al., 2015). This role in mouse skeletal development is consistent with genome-wide association studies implicating *SPAG17* in the control of human height (Kim et al., 2010; N'Diaye et al., 2011; Takeuchi et al., 2009; van der Valk et al., 2014; Weedon and Frayling, 2008; Weedon et al., 2008; Wood et al., 2014; Zhao et al., 2010). More recently, *SPAG17* variants were reported to cause primary ciliary dyskinesia in human patients (Andjelkovic et al., 2018). These variants are linked to male infertility owing to severe asthenozoospermia (Xu et al., 2018) and affect almost all stages of spermatogenesis in mice (Kazarian et al., 2018).

This study presents a phenotypic analysis of a new *Spag17* hypomorphic allele that we recovered from a 1-ethyl-1-nitrosourea (ENU) mutagenesis forward genetic screen. We named this allele primary ciliary dyskinesia only (*Pcdo*). Our data indicated that this *Spag17^Pcdo^* allele was not perinatal lethal, but homozygous mutants had a shorter lifespan and developed PCD phenotypes attributable to defects in the structural development of motile cilia. We saw no effects of the *Spag17^Pcdo^* variant on the development or function of primary cilia.

## RESULTS

### *Pcdo* is a nonsense allele of *Spag17*

We identified the *Pcdo* mutant in a mouse ENU mutagenesis forward genetic screen for recessive alleles disrupting organogenesis. ENU mutagenesis was performed as described previously (Stottmann and Beier, 2014), and we phenotyped the mice to recover mutant alleles at early postnatal stages as part of an experiment to look for mutants with abnormal forebrain development. As described further below, *Pcdo* mutants were initially identified by an enlarged head and distended lateral ventricles, easily visible upon gross dissection. Further histological examination confirmed the occurrence of hydrocephalus and abnormal accumulation of mucus in the respiratory passages. There was no evidence of heterotaxy or any other abnormal organ laterality.

In order to identify the causal variant in *Pcdo* mutants, we performed whole-exome sequencing on three phenotypic mutants (Table S1). We filtered for variants that were homozygous for the ‘alternative’ allele in each mutant not present in the dbSNP database (https://www.ncbi.nlm.nih.gov/snp/), to exclude strain polymorphisms predicted to have a ‘high’ or ‘moderate’ impact on protein coding and common to all three sequenced mutants. Only three genes of predicted ‘high’ impact met these criteria: plexin C1 (*Plxnc1*), Sfi1 homolog, spindle assembly associated (yeast) (*Sfi1*) and *Spag17*. A null allele of *Plxnc1* exists and does not have the same phenotype as the *Pcdo* mutants (Pasterkamp et al., 2003). *Sfi1* seems to be highly polymorphic, given that this appears routinely in similar analyses in our laboratory; therefore, we initially excluded this as a candidate. The null *Spag17* phenotype, however, was sufficiently similar to *Pcdo* to pursue as a candidate (Teves et al., 2013).

**Fig. 1.**
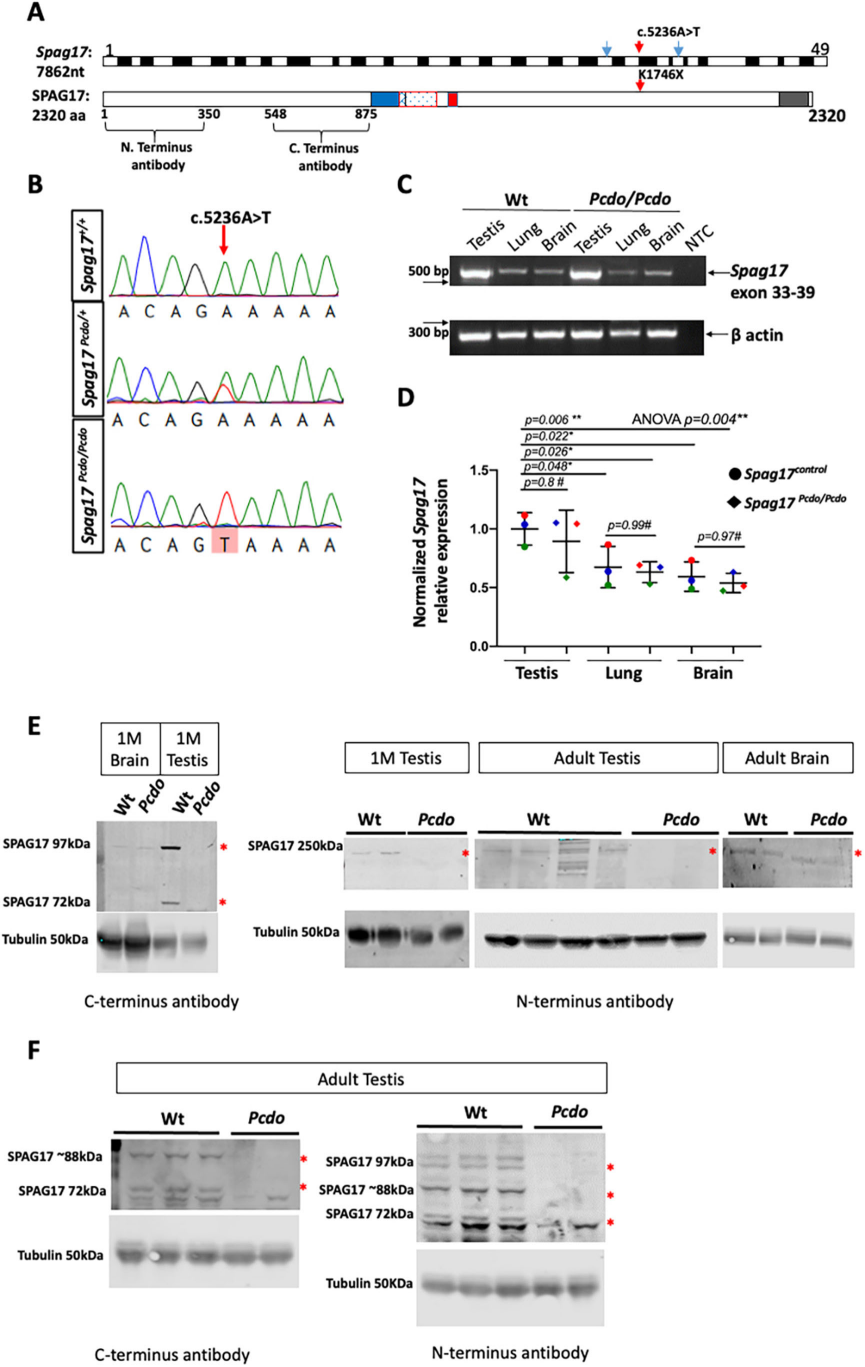
***Pcdo* is a nonsense allele of *Spag17*.** (A) The *Spag17* gene has 49 exons, and the position of the c.5236A>T ENU variant in exon 36 is indicated with red arrows (K1746* in the protein). Mouse SPAG17 has 2320 amino acids, and epitopes for the N-terminal and C-terminal SPAG17 antibodies are indicated. Blue arrows in the transcript indicate positions of the primers used in C. The mouse SPAG17 has coiled coil (blue), lysine-rich (blue dots), glycine-rich (red) and Pfam (gray) domains. (B) Sanger sequencing showing the A>T allele change in the *Spag17^Pcdo/Pcdo^* mutants. (C) Semi-quantitative RT-PCR of the *Spag17* exon 33-39 and β-actin loading control. (D) Quantification of *Spag17* expression in the testis, lung and brain tissues (relative to β-actin loading and normalized to testis wild-type levels: *n*=3 animals for each genotype; colors show littermates). (E) Western blotting with two different antibodies shows a lack of SPAG17 protein in *Pcdo* mutant testis compared with wild type and a decrease in the brain. (F) Analysis of the adult testis highlights another form of SPAG17 that is missing in the *Pcdo* mutants (red asterisks denote the loss or the reduction of SPAG17 isoforms in the mutant tissue).

The *Spag17-204* (ENSMUST00000164539) transcript has 49 exons and encodes for a full-length SPAG17 protein with 2320 amino acids (ENSMUSP00000134066; Fig. 1A). Another smaller splice variant, encoding a 97 kDa SPAG17 isoform, is found in the testis. This is known to be proteolytically processed during the process of spermatogenesis and sperm maturation to generate 72 and 28 kDa SPAG17 fragments (Silina et al., 2011; Zhang et al., 2005). The mouse SPAG17 protein has a small coiled coil domain, followed by two regions of compositional bias with lysine-rich and glycine-rich regions, followed by a PapD-like domain (flagellar-associated PapD-like, Pfam: PF14874). PapD-like domains are known to be highly conserved in mammals (Zhang et al., 2005; Fig. 1A).

Sanger sequencing of *Pcdo* DNA confirmed the A to T transversion at the start of exon 36, position c.5236, p.1746 (Fig. 1B). This mutation introduces a nonsense stop codon (TAA) instead of the wild-type lysine (K) amino acid at position 1746. The homozygous *Spag17^c.5236A>T^* mutation segregated completely with the mutant *Pcdo* phenotype and was confirmed by genotyping assays in more than 50 mutant animals over multiple generations to date*.* Semi-quantitative RT-PCR analysis of *Spag17* RNA using primers spanning exon 33 and 39 indicated high levels of *Spag17* transcript in the testis compared with very limited expression in the lungs and brain of 3-month-old animals (Fig. 1C). Quantification of the band intensity and normalization to a corresponding β-actin band were performed. We found that *Spag17* transcript was only marginally reduced in the *Pcdo/Pcdo* mutant tissues compared to wild type, suggesting that the early stop codon did not stimulate a nonsense-mediated decay response (Fig. 1D). We analyzed protein levels using two separate antibodies (Zhang et al., 2005). An antibody against an epitope in the C-terminus (Fig. 1A) reliably identified known isoforms with molecular weights of 97 and 72 kDa. Protein levels in the 1-month-old brain were low and similar in the wild type and mutant. In the testis at 1 month, both isoforms were expressed much more highly than in the brain, and they were completely missing in the mutant. With an antibody against an N-terminus epitope, we were able to identify the full-length 250 kDa species that was missing in the testis at both 1 month of age and in the adult mice. We saw only a slight reduction of this protein isoform in the brain (Fig. 1E). In addition to these isoforms, we also consistently saw an 88 kDa form with both antibodies in the adult testis. This seemed to be completely absent in mutant tissue (Fig. 1F). Given the more subtle effects on RNA, we suspect that this protein is degraded in the mutant tissues.

It was previously reported that the *Spag17* germline null animals suffered respiratory distress and died within the first 12 h of postnatal life due to defects in the respiratory motile cilia (Teves et al., 2013). In the *Spag17^Pcdo/Pcdo^* mutant animals, we observed frequent neonatal death within the first day of postnatal life (17.4%, *n*=4/23). Mutant animals that survived after the first day were viable to weaning age (*n*>37). *Spag17^Pcdo/Pcdo^* animals that were allowed to age beyond weaning experienced a shortened lifespan, with premature death at around 1-4 months of age (*n*=6/8). These animals were either found dead or were euthanized for humane reasons due to excessive morbidity. This is most probably attributable to severe hydrocephalus and chronic respiratory insufficiency. Approximately 25% (*n*=2/8) of *Spag17^Pcdo/Pcdo^* animals lived normally to an age of around 6 months before they were euthanized.

### Normal skeletal development and primary cilia in the *Pcdo* mutants

**Fig. 2.**
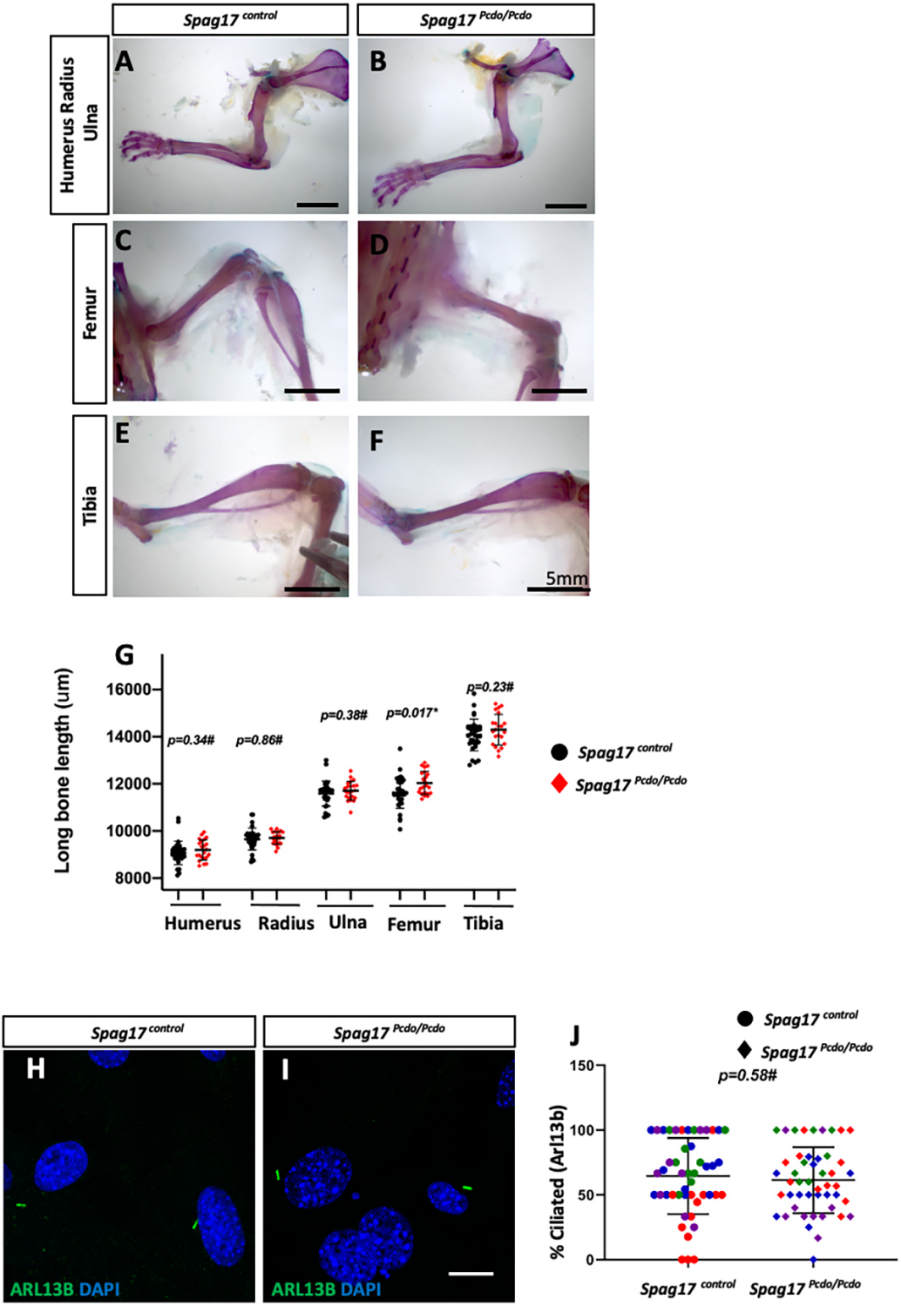
***Spag17^Pcdo^* does not interfere with skeletal development or primary ciliogenesis.** (A-F) Alizarin Red- and Alcian Blue-stained adult mouse upper limb skeleton (A,B), femur (C,D) and tibia (E,F) from control (A,C,E) and *Pcdo* mutants (B,D,F). (G) The lengths of the humerus, radius, ulna, femur and tibia are shown; *P=0.017 (# indicates no significant difference; *n*=16 and 11 for *Spag17^control^* and *Spag17^Pcdo/Pcdo^*, respectively). (H,I) Immunocytochemistry images of the *Spag17^Pcdo/+^* and *Spag17^Pcdo/Pcdo^* MEFs stained with ARL13B (green) and DAPI (blue). (J) The percentage of ciliated MEFs was not different between *Spag17^Pcdo^*^/+^ and *Spag17^Pcdo/Pcdo^* cells (*n*=4 animals for each genotype). Scatter plot in bars shows the mean and standard deviation; colors show littermates. Scale bars: 5 mm in A-F; 10 µm in I.

Previous reports indicated that SPAG17 is required for normal primary cilia function and for the growth and elongation of the long bones in mice (Teves et al., 2015). *SPAG17* has also been linked to height in human studies (N'Diaye et al., 2011; van der Valk et al., 2014; Weedon and Frayling, 2008; Weedon et al., 2008; Wood et al., 2014). We therefore investigated skeletal development in the *Spag17^control^* (*n*=16: 3 *Spag17^wt/wt^*, 13 *Spag17^Pcdo^*^/+^) and *Spag17^Pcdo/Pcdo^* (*n*=11) animals. We performed Alizarin Red and Alcian Blue skeletal preparations and measured the length of the long bones, including the humerus, ulna, radius (Fig. 2A,B), femur (Fig. 2C,D) and tibia (Fig. 2E,F). Statistical analysis (Student's unpaired *t*-test) did not detect a significant difference in any long bone lengths when comparing data between *Spag17^Pcdo/Pcdo^* mutants and control animals except for a slight increase in the *Spag17^Pcdo/Pcdo^* mutant femur length (Fig. 2G). The biological significance of this finding remains to be identified. To assay primary cilia structural development further, we generated MEFs from *Spag17^Pcdo^*^/+^ and *Spag17^Pcdo/Pcdo^* animals and performed immunocytochemistry for the ciliary membrane marker, ARL13B. The number of ciliated cells and the morphology of those cilia *in vitro* were not different in the *Spag17^Pcdo/Pcdo^* mutant cells when compared with control cells (Fig. 2H-J; 301 cells from *Spag17^control^* and 436 cells from *Spag17^Pcdo/Pcdo^*; four animals of each genotype were included in the analysis). These data indicate the *Pcdo* mutation does not affect the structure of primary cilia in MEFs or long bone development in the same way as total loss of *Spag17*.

### *Spag17^Pcdo^* mice show neonatal progressive hydrocephalus

**Fig. 3.**
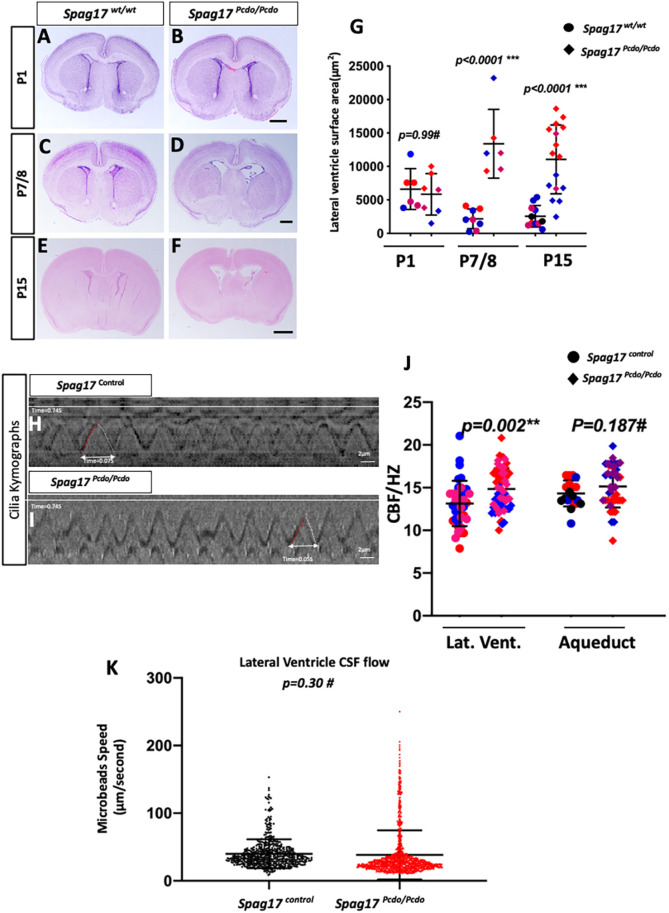
**Hydrocephalus was the first obvious phenotype in the *Spag17^Pcdo^* mutant line.** (A-F) Coronal brain sections from *Spag17^wt/wt^* and *Spag17^Pcdo/Pcdo^* littermates, showing the dilated lateral ventricles at P7 and older in the mutants. (G) Quantification is shown for multiple sections of three or more animals for each stage and genotype. (H,I) Representative kymographs from *Spag17^control^* (H) and *Spag17^Pcdo/Pcdo^* (I). (J) Scatter plots of the frequency of the motile cilia beating measurements obtained from lateral ventricle and aqueductal cilia. Lateral ventricle cilia are hyperkinetic and beat with a rhythm significantly faster than in the *Spag17* control animals (*n*=37 *Spag17^wt/wt^* lateral ventricle cilia and 69 *Spag17^Pcdo/Pcdo^* lateral ventricle mutant cilia obtained from three animals of each genotype; aqueduct, *n*=19 wild-type and 39 mutant cilia obtained from two wild-type and four mutant animals). (K) Scatter plots showing that there was no significant difference between flow speed of the flourescent microbeads in lateral ventricle brain slices (*n*=4 slices from three animals of each genotype). Scale bars: 500 µm in B,D; 1 mm in F; 2 µm in H,I.

Hydrocephalus is a prevalent phenotype in PCD mouse models. Likewise, *Spag17^Pcdo/Pcdo^* mutant animals developed a fully penetrant, mildly progressive hydrocephalus. The hydrocephalus was first obvious shortly after birth and before the beginning of the second postnatal week (Fig. 3A-F). Measurement of the lateral ventricle area from mutants compared with wild-type control animals showed no difference at postnatal day (P)1 (Fig. 3A,B; *n*=3 wild type and 3 mutant). This measurement was significantly increased in the mutants by P7 (Fig. 3C,D; *n*=3 wild type and 3 mutant) and remained significantly higher at P15 (Fig. 3E-G; *n*=4 wild type and 3 mutant). No masses of abnormal growths indicative of obstructive hydrocephalus were observed within the ventricular system (Fig. S1). However, we did observe severe overt intracerebroventricular and subarachnoid hemorrhage in a small subset of *Spag17^Pcdo/Pcdo^* mutant animals (*n*=3/32). As expected, this led to blood clots and obstruction of the ventricular system at various points and, ultimately, severe obstructive hydrocephalus and fatality (Fig. S2).

The hydrocephalus in the motile cilia models has usually been attributed to perturbed flow of cerebrospinal fluid (CSF) towards the next most caudal opening within the cerebroventricular system. A highly conserved function of the ependymal motile cilia lining the brain ventricles seems essential for directing this CSF flow. Given the *Pcdo* phenotypes, we hypothesized that ciliary beating might be severely compromised in *Spag17* animals (Teves et al., 2013). We examined this using *ex vivo* high-speed video microscopy at P4 (Movies 1 and 2). We showed previously that the ependymal motile cilia follow a spatiotemporal pattern of development in the developing forebrain and lateral ventricle, and ependymal motile ciliogenesis was seen along the medial wall of the lateral ventricle at ∼P0, as opposed to the lateral wall, where ependymal ciliogenesis starts towards the end of the first postnatal week (Abdelhamed et al., 2018). We recorded the ciliary beating in both the lateral ventricle and the aqueduct. Surprisingly, we did not detect any reduction in the ciliary beat frequency in any of the areas we measured (Fig. 3H-J). On the contrary, we observed that the *Pcdo* mutant motile cilia of the medial walls of the lateral ventricle were slightly hyperkinetic (Fig. 3J). Detailed analysis of the kymographs of the beating cilia showed altered waveforms, as indicated by a shorter interval between forward strokes in *Pcdo/Pcdo* mutants (Fig. 3H,I). A quantification of this showed that the mutant cilia were beating at a rate significantly higher than control cilia in the lateral ventricle (*P*=0.002; Fig. 3J). Interestingly, kymographs of the beating cilia and analysis of the cilia beat frequency did not detect any significant difference in the aqueduct cilia (*P*=0.189; Fig. 3J). We also measured CSF localized flow with fluorescent microbeads introduced *ex vivo* into the forebrain slice immediately before the video microscopy recording. This analysis did not show abnormalities in flow speed in isolated forebrain lateral ventricle slices (*P*=0.30; Fig. 3K; Movies 3 and 4). These data are consistent with our conclusion that the *Pcdo* K1746* allele of *Spag17* is a hypomorph, in comparison to the previously reported *Spag17* null allele, which develops paralyzed cilia that affect mucociliary clearance and neonatal survival (Teves et al., 2013).

### *Pcdo* aqueductal stenosis disrupts bulk CSF flow

**Fig. 4.**
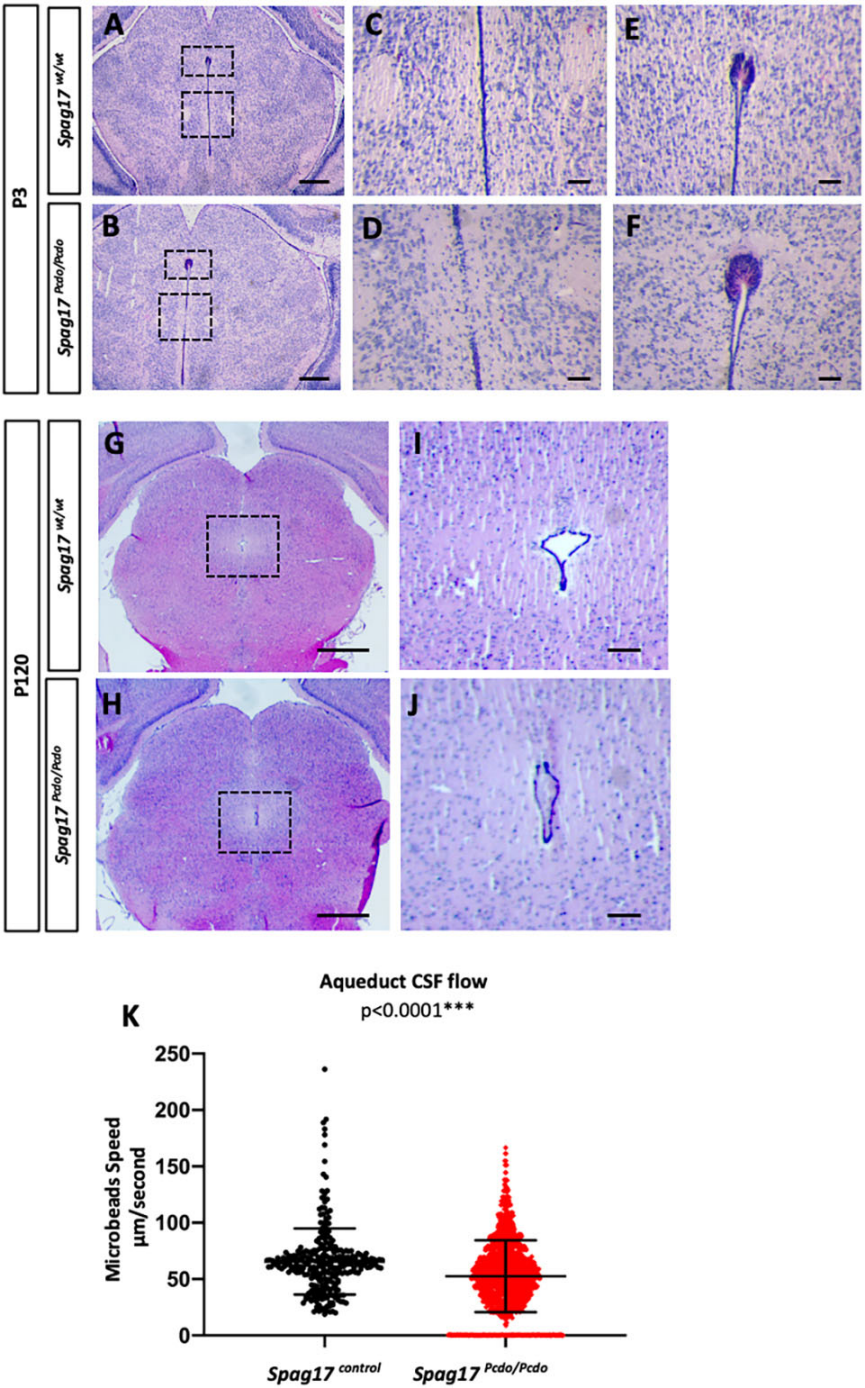
**Aqueduct stenosis led to disturbed CSF flow and hydrocephalus in the *Spag17^Pcdo/Pcdo^* mice.** (A,B) Hematoxylin and Eosin-stained coronal sections through the P3 aqueduct from *Spag17^wt/wt^* (A) and *Spag17^Pcdo/Pcdo^* (B). (C-F) Boxed areas of magnified aqueduct lumen (C,D) and SCO (E,F), as indicated in A,B. (G-J) Coronal P120 aqueduct sections from *Spag17^wt/wt^* (G) and *Spag17^Pcdo/Pcdo^* (H); boxed areas are magnified in I,J. Scale bars: 500 µm in A,B,G,H; 100 µm in C-F,I,J. (K) Scatter plots showing the speed of the moving beads inside the aqueduct (data collected from approximately four slices from two control and four mutant animals).

The aqueduct of Sylvius is a narrow channel for CSF flow connecting the third ventricle to the fourth ventricle. This channel is obstructed in mouse models with deficits in primary cilia (Town et al., 2008) and in motile ciliopathy attributable to collapse and fusion of the walls of the ventricle (Ibañez-Tallon et al., 2004). Histological characterization of the aqueduct at P4 from *Pcdo* mutant animals indicated fusion of the ependymal lining at multiple points of the aqueduct (Fig. 4A-D; *n*=3 wild type and 3 mutants). We also noted some enlargement of the subcommissural organ (SCO) in the *Spag17^Pcdo/Pcdo^* mutant animals (Fig. 4E,F). Later in development, the mutant aqueduct appeared collapsed and shrunken (Fig. 4G-J; *n*=4 wild type and 4 mutants). The SCO is an area of highly differentiated ependyma located in the dorsocaudal region of the third ventricle at the entrance to the aqueduct of Sylvius and is well known for secreting high molecular weight glycoproteins necessary for CSF flow and circulation (Rodríguez et al., 1987). Hydrocephalus is a common feature in animal models with loss or defective development of the SCO (Blackshear et al., 2003; Cao and Wu, 2015; Sakakibara et al., 2002; Stoykova et al., 1996). Collectively, these studies and others suggest that the secretory activity of the SCO is responsible, at least in part, for the maintenance of an open aqueduct. We suspected that aqueductal stenosis could cause localized CSF flow abnormalities and hydrocephalus in the *Spag17^Pcdo/Pcdo^* mutants. We tested this with video microscopy and fluorescent microbeads in the intact aqueductal lumen (Movies 5 and 6). We saw that the aqueductal stenosis significantly affected the directionality and speed of the moving beads in the P4 mutant aqueduct (Fig. 4K; *P*<0.0001; *n*=2 control and 4 mutant animals). We conclude from these data that CSF bulk flow is extremely disturbed along with aqueductal stenosis and that this is what ultimately leads to the hydrocephalus in the *Pcdo* mutants.

### *Pcdo* mutants show other PCD-related phenotypes

**Fig. 5.**
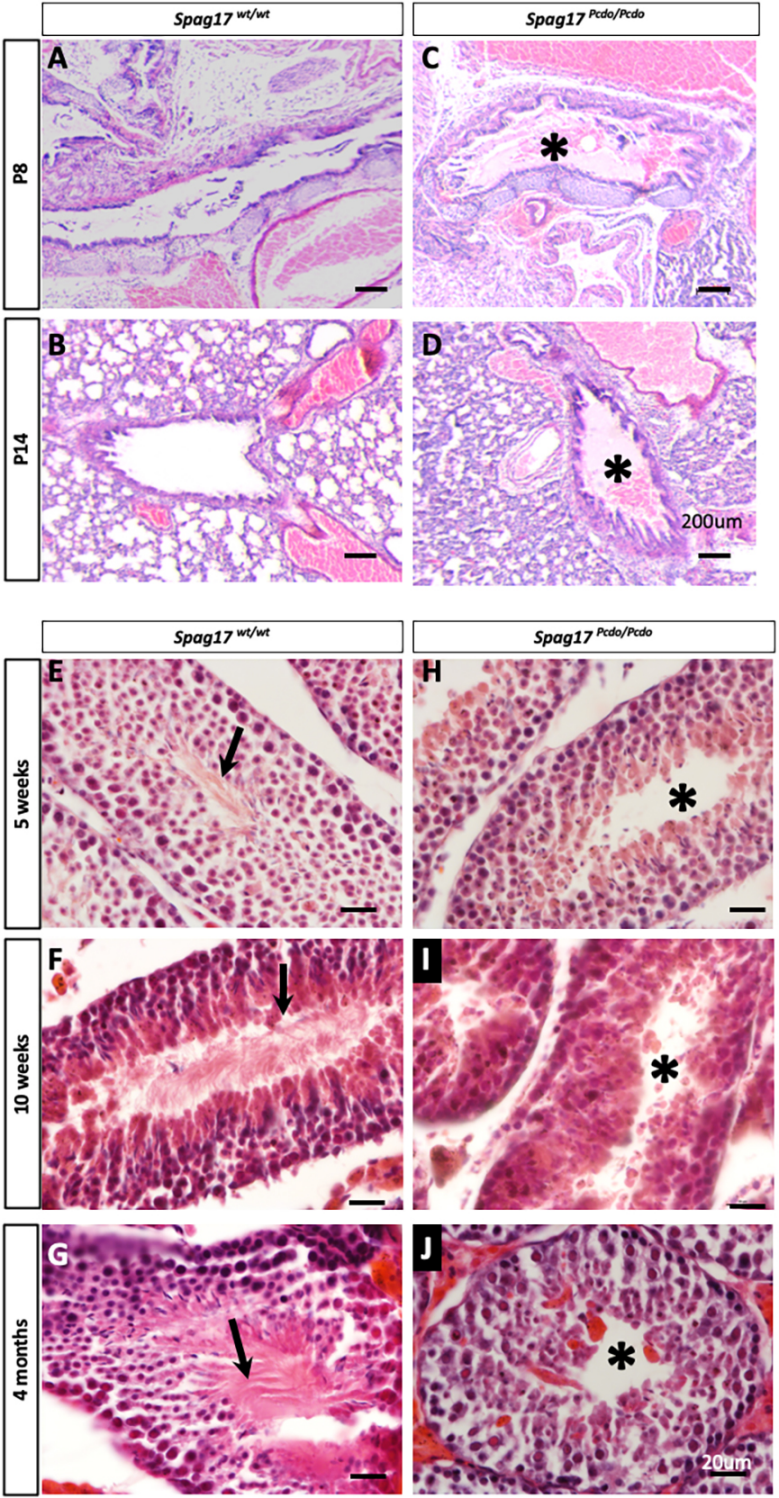
***Spag17^Pcdo/Pcdo^* mutants are generally viable and develop PCD phenotypes.** (A-D) Histological analysis of *Spag17^wt/wt^* (A,B) and *Spag17^Pcdo/Pcdo^* (C,D) trachea and lung showing mucus accumulation (asterisks) in the trachea and main bronchus in the mutant animals at P8 (A,C) and P14 (B,D). (E-J) Seminiferous tubules of the testis at 5 weeks (E,H), 10 weeks (F,I) and 4 months (G,J) from *Spag17^wt/wt^* (E-G) and *Spag17^Pcdo/Pcdo^* mutants (H-J). In E-G, arrows in the *Spag17^wt/wt^* sections point to a mature sperm flagellum. The *Spag17^Pcdo/Pcdo^* mutants lack a sperm flagellum at all stages presented, indicated by asterisks in H-J. Scale bars: 200 µm in A-D; 20 µm in E-J.

Gross anatomical assessment of the visceral organs in *Spag17^Pcdo/Pcdo^* mutants showed that organ laterality was unaffected in this model in all animals examined (Fig. S3; *n*=5 animals each genotype)*.* Histological and gross anatomical examination of the lung did not show any right or left lung isomerism, and gross lung development was not affected in the *Spag17^Pcdo/Pcdo^* mutants (Fig. S3; *n*=18 control and 15 mutants). However, detailed histological examination of the lungs and trachea from P8 and P14 *Spag17^Pcdo/Pcdo^* mutants showed abnormal accumulation of homogenous eosinophilic material filling the upper respiratory passages, the trachea and main bronchi, indicative of mucus accumulation, most probably attributable to defective mucociliary clearance (Fig. 5A-D; *n*=6 control and 6/7 mutants).

Another common PCD phenotype is male infertility. All male *Spag17^Pcdo/Pcdo^* animals we tested (*n*=4) were infertile and were unable to produce any litters when mated with wild-type control female mice (when paired twice for 5 weeks at each attempt, *n*=8). Conventional histological analysis of the testis was performed at 5 weeks (*n*=4 wild type, 3 mutants), 10 weeks (*n*=3 wild type, 3 mutants) and 4 months of age (*n*=3 wild type, 3 mutants). In all stages of testicular development we examined, the *Spag17^Pcdo/Pcdo^* mutant seminiferous tubules were devoid of any sperm, with a clear lumen, whereas all control sections showed evidence of normal spermatogenesis, with mature sperm observed in the center of the seminiferous tubules (Fig. 5E-J). No clear difference was noted at the level of the germ cells or the number or morphology of developing spermatocytes (Fig. 5E-J). The round spermatids failed to mature fully and failed to produce flagella (Fig. 5E-J). This indicates that the processes involved in spermiogenesis, including development of the sperm flagellum, require SPAG17.

### Differential requirements of *Spag17* for motile ciliogenesis in the brain, lung and sperm flagellum

**Fig. 6.**
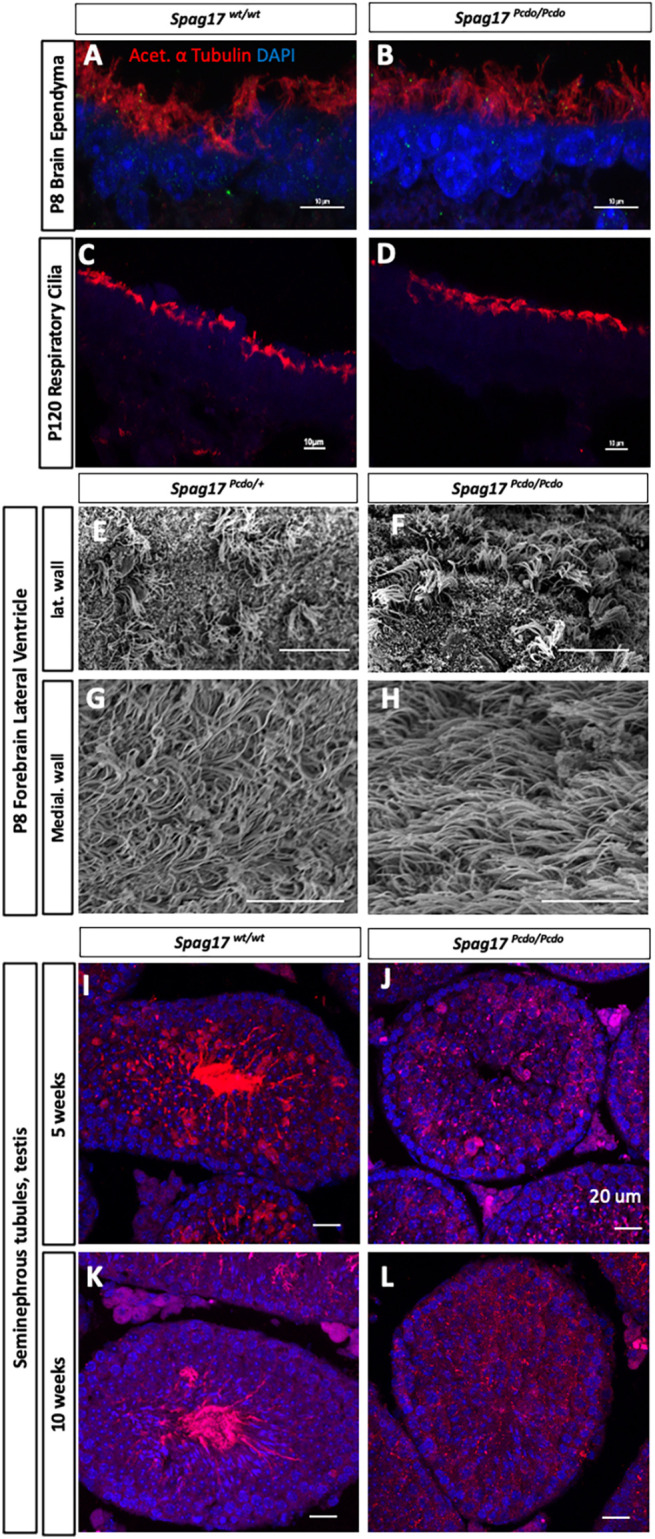
**SPAG17 is essential for development of the sperm flagellum but not for initiation of motile ciliogenesis in the brain or the respiratory tract.** (A-D) Forebrain ependymal (A,B) and tracheal epithelial (C,D) cells of *Spag17^control^* (A,C) and *Spag17^Pcdo/Pcdo^* (B,D) animals stained for the ciliary axoneme with acetylated α-tubulin (red) and DAPI to stain nuclei (blue). (E-H) SEM images of the lateral wall (E,F) and medial wall (G,H) of the P8 forebrain ependymal cilia. No clear differences are observed in the overall morphology of the ependymal or the respiratory motile cilia. (I-L) Seminiferous tubules of *Spag17^wt/wt^* (I,K) and *Spag17^Pcdo/Pcdo^* (J,L) animals stained for acetylated α-tubulin (red) and DAPI (blue). No staining of the cilia can be detected in the *Spag17^Pcdo/Pcdo^* mutants (J,L) compared with *Spag17^wt/wt^* (I,K). Scale bars: 10 µm in A-H; 20 µm in I-L.

Given the functional deficits we observed in the motile cilia, we performed a morphological analysis of the motile ciliary structure. We first performed immunohistochemical analysis with the acetylated α-tubulin antibody to stain the ciliary axoneme. No apparent morphological differences were detected in the *Spag17^Pcdo/Pcdo^* mutant ependymal and/or respiratory cilia when compared with wild-type control cilia (Fig. 6A-D). We saw similar results with a more stringent scanning electron microscopy (SEM) analysis. The abundance of cilia and length of cilia appeared comparable in ependymal cilia lining the medial and lateral walls of the forebrain lateral ventricle, indicating that the *Spag17^Pcdo^* allele does not perturb production of motile cilia in either the brain ependymal cells or the respiratory epithelial cells (Fig. 6E-H; *n*=4 wild type and 4 mutants). By contrast, *Spag17^Pcdo/Pcdo^* mice showed deficits in formation of the sperm flagellum. Consistent with the histological analysis above, immunohistochemical staining of testicular sections from *Spag17^Pcdo/Pcdo^* mutants showed a total lack of acetylated α-tubulin staining at 5 and 10 weeks of age in comparison to the age-matched controls, in which we observed clear and pronounced acetylated α-tubulin staining (Fig. 6I-L; *n*=3 wild type and 3 mutants for each age).

### Distinct ultrastructural defects in the *Spag17^Pcdo^* mutant respiratory and brain ependymal motile cilia

**Fig. 7.**
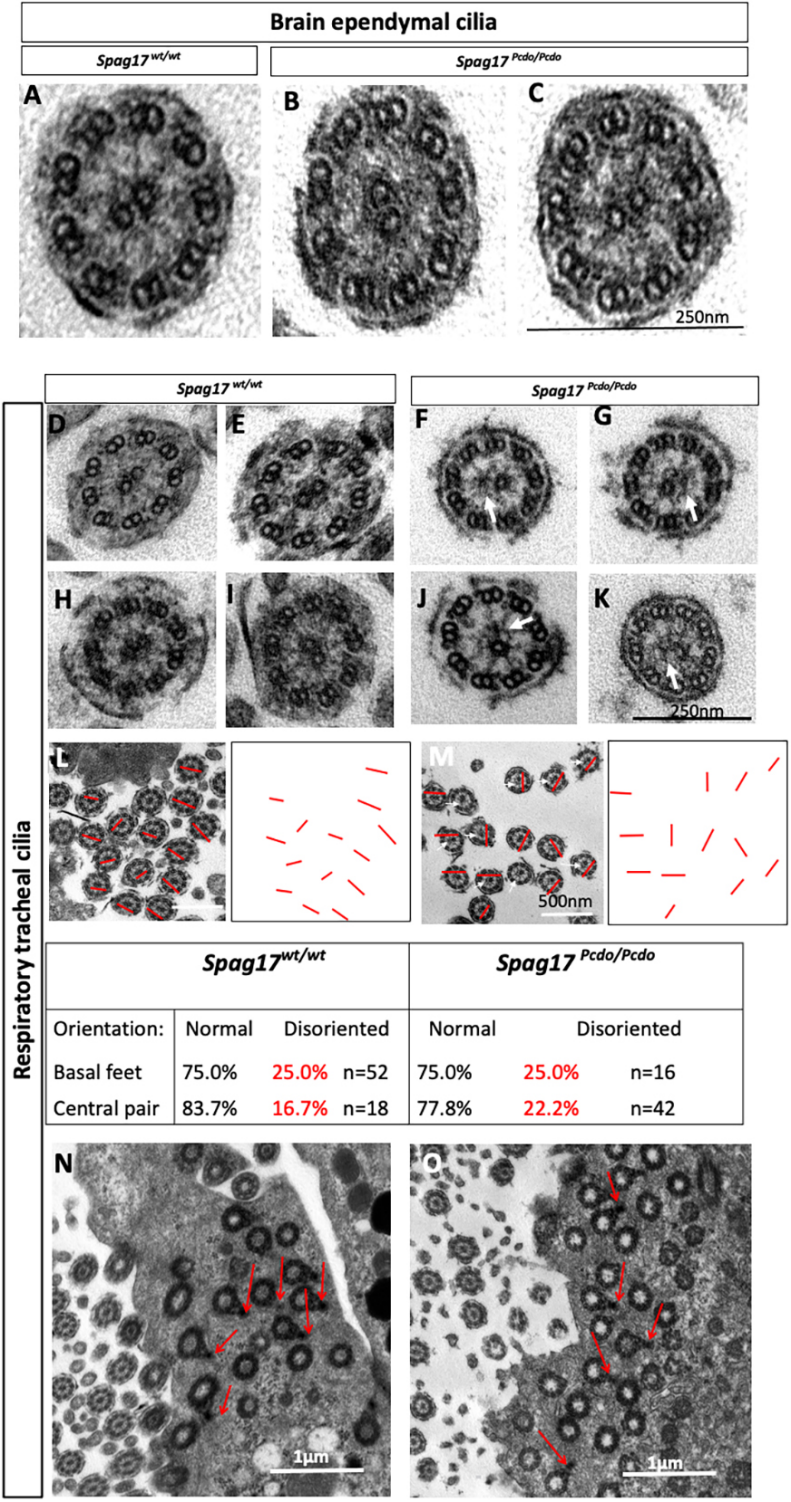
**SPAG17 is essential for central pair development and stability in the respiratory epithelial cilia.** (A-C) TEM cross-section of the ependymal cilia from *Spag17^wt/wt^* (A) and *Spag17^Pcdo/Pcdo^* (B,C) showing no clear ultrastructural defects in the mutant ependymal cilia (*n*=58 cilia from wild-type and *n*=64 cilia from mutant cross-sections; three different specimens for each). (D-K) Higher magnification of the respiratory cilia axoneme cross-sections, showing lack of (arrows in F,G) or abnormal (arrows in J,K) C1 microtubule structure of the central pair apparatus in the *Spag17^Pcdo/Pcdo^* mutants (F,G,J,K; *n*=2 animals) compared with cross-sections of wild-type (D,E,H,I; *n*=2 animals) respiratory cilia. (L,M) TEM images of the respiratory epithelium of *Spag17^wt/wt^* (L) and *Spag17^Pcdo/Pcdo^* (M) animals (red lines indicate the axis of orientation of the central pair; the *Spag17^Pcdo/Pcdo^* mutant central pair(s) were abnormally oriented in different directions; thin white arrows in M point to cross-sections of cilia that have no C1 microtubule). (N,O) Basal processes of the basal bodies were normally and equally aligned in the respiratory epithelial cells from *Spag17^wt/wt^* (N) and *Spag17^Pcdo/Pcdo^* (O) mutants (red arrows). Scale bars: 250 nm in C,K; 500 nm in L,M; 1 µm in N,O.

SPAG17 is a structural protein within cilia essential for development of the C1a process of the C1 microtubule structure of the central pair apparatus (Zhang et al., 2005). We therefore investigated the ultrastructure of the ependymal and respiratory motile cilia using transmission electron microscopy (TEM). Cross-sections of ependymal cilia appeared fairly normal, with no clear defects in either the structure or the orientation of the central pair apparatus or other motility structures, such as inner or outer dynein arms (Fig. 7A-C). This is consistent with the limited functional disturbance in ependymal ciliary beat frequency and the undisturbed localized (intraventricular) CSF flow observed in this model. By contrast, TEM analysis of the cross-sections of respiratory cilia showed predominantly abnormal respiratory tracheal cilia in the *Spag17^Pcdo^* mutants. Two types of related deformities can be observed clearly. First, mutant respiratory cilia lack one of the central pair microtubule singlets in a subset of the mutant cilia, with the resulting appearance of ‘9+1’ microtubule organization (Fig. 7D-G). Second, most of the remaining cilia in the mutant trachea have an abnormal central pair apparatus, with one of the central pair singlets appearing smaller or incomplete compared with wild type (Fig. 7H-K). Rotational polarity of the motile cilia axoneme is dependent on the central pair orientation (Kunimoto et al., 2012). Orientation of the axonemal central pair of microtubules was measured by drawing a line through the central pair and measuring the angle of the line with respect to the 0-180° angle arbitrarily set for the first line drawn, as previously described (Hegan et al., 2015; Kunimoto et al., 2012). The deviation from this angle was larger in the *Pcdo* mutant cilia, and 22.2% of the *Spag17^Pcdo^* mutant central pairs (*n*=42) in the respiratory cilia were not properly aligned (22.2% versus 16.7% in wild type, *n*=18; Fig. 7L,M). This indicates that rotational planar cell polarity of the central pair was perturbed in the *Spag17^Pcdo/Pcdo^* mutant respiratory cilia. SPAG17 is not only essential for central pair development but could also be indispensable for establishing the central pair rotational polarity. The observed degree of central pair disorientation could have a strong negative influence on the highly coordinated ciliary beating and thereby contribute to impaired directional fluid flow and mucociliary clearance in the lung.

The observed central pair orientation defect prompted us also to assay the orientation of the basal body basal feet in the mutant respiratory cilia compared with wild type. The directionality of ciliary beating is determined by the orientation of the basal feet of the basal bodies, which are usually positioned at the fourth, fifth and sixth circularly arranged triplets of microtubules within the basal bodies. The basal feet associated with the basal bodies are usually oriented in the direction of the effective stroke of the ciliary beating, and this is controlled by the planar cell polarity pathway (Boisvieux-Ulrich et al., 1985; Gibbons, 1961; Kunimoto et al., 2012). However, we found that in the *Spag17^Pcdo/Pcdo^* mutant cilia (*n*=16 cilia), the basal feet of the basal body appeared to be aligned normally when compared with wild-type control cilia (*n*=52 cilia; Fig. 7N,O). We did not observe further defects in other structures required for ciliary motility, such as inner and outer dynein arms.

Altogether, our data indicate that there is a differential requirement for SPAG17 among the different cell types that produce motile cilia. Based on the phenotypes we observe in the testis, lung and brain, we conclude that SPAG17 is crucial for initiation and formation of the sperm flagellum, whereas it has a more specific role (limited to central pair apparatus and C1a projection development and orientation) during structural development of the respiratory cilia and an even more limited role during differentiation and maturation of cilia of the brain ependymal cells.

## DISCUSSION

### *Pcdo* is an allele of *Spag17* revealing tissue-specific effects on SPAG17 translation

In this study, we present a detailed characterization of the *Pcdo* mutant we recovered from an ENU forward genetic screen and identified as a nonsense allele of *Spag17* (Fig. 1A,B). The *Pcdo* variant is a c.5236A>T nonsense mutation predicted to introduce a premature stop codon at position 1746 of the 2320 amino acid SPAG17 protein. Significantly higher levels of *Spag17* RNA were expressed in the testis in comparison to the lungs and brain tissues of the adult mice. These data are consistent with previous *Spag17* expression analyses (Zhang et al., 2005). The *Pcdo* mutation resulted in only a slight reduction in RNA expression in mutant tissues (Fig. 1C,D), indicating that the *Pcdo* mutation does not induce nonsense-mediated decay in any tissue examined. Protein analyses of mutant tissue samples from testis and brain indicated that the *Pcdo* mutation leads to the loss of multiple SPAG17 protein isoforms in testis but not in brain, although the ENU-induced variant is common to the full-length isoform of *Spag17* known to be expressed in testis, brain and lung. Our analysis also identified very low levels of the SPAG17 97 kDa isoform in the brain, which did not seem to be affected in the *Pcdo* mutants. The precise mechanism leading to loss of the SPAG17 protein in the *Pcdo* mutants remains to be determined. Similar observations have been documented in studies of another motile cilia gene, adenylate kinase 7 (*Ak7*), in which mutation leads to loss of protein in the testis but not in the respiratory cilia (Lorès et al., 2018). Our data indicate that proteins expressed in both motile cilia and sperm flagella might have tissue-specific properties in each of these two highly related organelles. This might be attributable to the major differences between sperm flagella and simple motile cilia in the brain and the respiratory epithelium.

### Mild phenotypes observed in *Pcdo* mutant animals indicate that *Pcdo* is a hypomorphic allele of *Spag17*

*Spag17^Pcdo/Pcdo^* mice developed early postnatal communicating hydrocephalus and had accumulation of mucus in the respiratory passages, and male mutants were infertile. Although the majority of *Pcdo* mutant animals survived weaning, they had a shortened lifespan compared with controls. All these phenotypes are consistent with deficits of the motile cilia in the *Pcdo* mutants. Despite these indicators of deficits of the motile cilia, beating and fluid flow were not reduced in the mutant lateral ventricle. Instead, we determined the cause of the hydrocephalus to be progressive aqueductal stenosis leading to CSF fluid turbulence and disrupted ventricular bulk flow. *Pcdo* mutant mice did not have obvious defects in formation of the primary cilia in the MEFs that we examined directly. Furthermore, the *Pcdo* mutants did not have the hallmark phenotypes of mouse models lacking primary cilia (e.g. Marszalek et al., 1999). These findings that *Pcdo* mutants develop milder gross anatomical and ciliary motility phenotypes and survive longer than the *Spag17* knockout mouse phenotypes (Teves et al., 2013) support our conclusion that *Pcdo* is a hypomorphic allele of *Spag17*. Previous data have shown some variability in PCD phenotypes based on genetic background; therefore, we cannot exclude the idea that this might contribute to the discrepancy with published results. Our experiments were performed in mice of a mixed C57BL/6J and FVB/NJ background, and the previous report of the null allele used mice of a mixed C57BL/6J and 129S4/SvJ background. However, at least one report has suggested that these phenotypes tend to be less severe on the 129 background (Finn et al., 2014). Taken together, we do not favor a model wherein the differences in phenotype are largely attributable to effects of the genetic background, but this should be considered.

### Motile and primary ciliopathies can overlap in the same model

Motile and primary ciliopathies have long been considered as two distinct dysfunctions of two related but functionally different organelles, the motile and nonmotile cilia, respectively. However, recent advances and further characterization of ciliopathy mutants have indicated that motile and primary ciliopathies can overlap in the same animal model and/or human patients (Bukowy-Bieryłło et al., 2013; Ferkol and Leigh, 2012; Fliegauf et al., 2007; Moore et al., 2006). Characterization of the *Spag17* knockout mice provided new evidence that motile and primary ciliopathies can overlap or develop in the same model organism (Teves et al., 2013, 2015). *SPAG17* mutations are reported to cause human PCD (Andjelkovic et al., 2018), and variants in the gene have been linked to human height (Kim et al., 2010; Takeuchi et al., 2009; Weedon and Frayling, 2008; Weedon et al., 2008; Zhao et al., 2010) and multiple body measurements in goats (Zhang et al., 2019). Interestingly, combined missense changes in *SPAG17* and *WDR35* cause complex neurodevelopmental malformations and skeletal cranioectodermal dysplasia, a primary ciliopathy (Córdova-Fletes et al., 2018). These studies confirm that *Spag17* has a role in the development and function of both motile and immotile primary cilia, but the effects differ based on the precise allele in question.

### Similar to other mutants in central pair genes, *Pcdo* mutants have no laterality defects

A hallmark of a central pair PCD ciliopathy is the absence of laterality defects, subtle ciliary beating abnormalities, and unequivocal ultrastructural defects of the ciliary axoneme. Vertebrate organ asymmetry is well known to require the nodal cilia that generate leftward flow across the node. These cilia are motile because they express inner and outer dynein arms but do not have a central pair apparatus. It is therefore intriguing to see that almost no central pair protein mutants, including this allele of *Spag17*, develop laterality defects (Cindrić et al., 2020; Davy and Robinson, 2003; Lechtreck et al., 2008). However, mutants of the dynein complexes consistently develop various degrees of laterality defects, including dextrocardia and heterotaxy (Abdelhamed et al., 2018; Bartoloni et al., 2002; Dougherty et al., 2016; Loges et al., 2018; Ta-Shma et al., 2018). The lack of laterality phenotypes in the *Spag17^Pcdo/Pcdo^* mutants further supports the model that central pair proteins, including *Spag17*, are dispensable for the development of organ asymmetry and body laterality.

### Ependymal cilia show normal ultrastructure and minimal beating defects

The main function of the ependymal cilia is to beat and generate CSF flow. The forebrain ependymal cilia in the *Spag17^Pcdo^* mutant have no apparent ultrastructural abnormalities, and the ciliary beating frequency was unaffected in the ependymal cilia of the aqueduct (Fig. 3J). Surprisingly, a subset of the ependymal cilia on the medial wall of the lateral ventricle in the *Spag17^Pcdo/Pcdo^* mutants are hyperkinetic and beat with a rate significantly higher than that of the control cilia (Fig. 3J). Our data suggest, however, that this hyperkinetic beating does not disrupt the local cilia-mediated flow in the acute *ex vivo* isolated medial wall forebrain slice (Fig. 3K). It is possible that this could have a more prominent effect *in vivo*, where there are more limiting space constraints. Alternatively, the lack of an effect on the structural development and function of the ependymal cilia could be attributable to the expression of near-normal levels of SPAG17 proteins in brain ependymal cells (Fig. 1). Interestingly, the hyperkinetic cilia phenotype in the *Spag17^Pcdo/Pcdo^* mutants is consistent with other motile cilia mutant phenotypes that have normal motile cilia ultrastructure but hyperkinetic cilia, such as *DNAH11* PCD patients (Bartoloni et al., 2002; Dougherty et al., 2016; Knowles et al., 2012; Pifferi et al., 2010; Schwabe et al., 2008) and a mouse model (Handel and Kennedy, 1984). Assessment of this beating pattern of the CSF flow *in vivo* would be a better indicator of the effect of the hyperkinetic cilia on the fluid flow and development of hydrocephalus. This would also be helpful to explain whether the hyperkinetic cilia phenotype is causing the hydrocephalus in this model directly or whether other mechanisms are responsible.

### The hydrocephalus in the *Spag17^Pcdo/Pcdo^* mutants is attributable to aqueductal stenosis

The aqueduct of Sylvius in the *Pcdo* mutants appeared stenotic, with collapsed walls, and was associated with a slight enlargement of the SCO (Fig. 4). In other models, patency of the aqueduct is compromised in the presence of a dysfunctional SCO or SCO ependymal cilia (Pérez-Fígares et al., 2001; Swiderski et al., 2012), leading to aqueductal stenosis or occlusion and noncommunicating hydrocephalus. Ciliary beating function and directionality were defective when assayed in the intact aqueduct lumen. The stenosis could have led to a mechanical constraint on the ciliary beating and thus to significantly reduced flow of CSF into the fourth ventricle. We concluded that *Pcdo* mutants had communicating hydrocephalus because the aqueduct was stenotic and not completely occluded, indicative of a potentially milder dysfunction of the SCO.

### Tissue-specific effects of reduced *Spag17* function

Surprisingly, we noted severely defective structure of the respiratory motile cilia consistent with a central pair protein insult. The *Spag17^Pcdo^* mutant cilia either lack one of the central microtubule pair or have an abnormal central pair structure. The orientation of the central pair also appeared not to align in one direction, indicative of disorganization of ciliary central pair polarity (Fig. 7L,M). This is expected to interfere with a directional fluid flow in the respiratory passages and lead to mucus accumulation and recurrent infection. The axis of polarity is controlled by signaling and mechanical cues. The mechanical cue is a cilia-generated fluid flow (Mitchell et al., 2007). The intense mucus accumulation observed in the *Spag17^Pcdo/Pcdo^* mutants indicates that ciliary motility in the lung is defective. This is consistent with the structural defects and is likely to contribute significantly to increased morbidity and the shortened lifespan in these mutants. We should note a limitation to our ultrastructural studies. We were able to analyze multiple cilia, but these were from a relatively limited set of animals. Current world-wide experimental conditions are challenging our ability to collaborate in order to obtain more robust sample sizes for this portion of the study. Moreover, development of the sperm flagellum was completely inhibited in the *Pcdo* mutants, and no mature sperm with a flagellum can be detected at any postnatal stage of testicular development (Figs 5E-J and 6I-L).

Altogether, our data indicate that SPAG17 is necessary for sperm flagellum development and important for proper assembly of the respiratory motile cilia central pair apparatus but is not required in a similar manner in the development of ependymal cilia. *Spag17* is highly expressed in the testis, with moderate expression levels in the lungs, and the lowest levels observed in the brain (Fig. 1C,D). We note a correlation between the basal *Spag17* expression levels and the downstream results of *Pcdo* mutation in the different motile cilia examined. Spermatocytes and round spermatids seem to have an absolute requirement for SPAG17. Although other cell types of motile cilia can be more tolerant of *Pcdo* mutation, the respiratory cilia cannot compensate for the mild reduction in *Spag17*. At the protein level, SPAG17 is known to interact with other central pair proteins, such as SPAG16 and SPAG6, and to form a complex at the C1a microtubule projections (Fu et al., 2019; Wargo et al., 2005; Zhang et al., 2005; Zhao et al., 2019). One explanation of this finding is that the minimal levels of SPAG17 produced in the *Pcdo* mutant ependymal cells were sufficient to facilitate the interaction with other proteins and to form the C1a projection, but much more SPAG17 was needed in the respiratory cilia to form these protein complexes and thereby form the correct central pairs of the motile respiratory cilia. All isoforms of SPAG17 were completely abolished in the *Pcdo* mutant testis; therefore, it seems that the C1a projection interactome was unable to form, and this led to complete failure of formation of the sperm flagellum. Several testis-specific proteasome/ubiquitin enzymes have been reported in rodents (Mochida et al., 2000; Nishito et al., 2006) and humans (Mitchell et al., 1991; Uechi et al., 2014). It is therefore also possible that the mutant SPAG17 protein is degraded in the testis by a sperm-specific proteasome-mediated quality control mechanism (Morales et al., 2003; Sutovsky, 2011; Zimmerman and Sutovsky, 2009). Other possibilities include the simple explanation that not all tissues construct axonemes from the same protein set, and SPAG17 is functionally compensated for in cilia outside the testis. Altogether, these data are consistent with recent reports that effects of specific gene mutations on protein translation and thereby function might differ dramatically between various cell types expressing the same allele (Lucas et al., 2019).

In summary, this study describes a new allele of *Spag17* that encodes the C1a projection SPAG17 protein. The *Pcdo* allele recapitulated most PCD phenotypes previously observed in other models due to central pair abnormalities. These are often the most difficult to diagnose in humans owing to lack of laterality phenotypes, only subtle beating defects and largely undisturbed ciliary ultrastructure (Edelbusch et al., 2017). We suggest that the *Spag17^Pcdo^* allele is a very useful model for studying the pathogenesis and molecular mechanisms of human PCD subsequent to central pair defects.

## MATERIALS AND METHODS

### ENU mutagenesis and recovery of *Pcdo* mutants

ENU mutagenesis was performed as described by Herron et al. (2002) and Stottmann and Beier (2014). Briefly, 6- to 8-week-old C57BL/6 *Mus musculus* males (G0 males) were injected intraperitoneally with three weekly fractioned doses of ENU and then bred to FVB *Mus musculus* females (The Jackson Laboratory) to generate G1 heterozygous carrier males. These G1 males were then outcrossed to FVB females to generate G2 potentially heterozygous carrier females. These G2 females were backcrossed to their respective G1 male parent to generate G3 embryos or pups, which were screened for organogenesis phenotypes. The *Pcdo* mutant mice were first identified with dilated brain ventricles and hydrocephalus. All animals were maintained through a protocol approved by the Cincinnati Children's Hospital Medical Center IACUC committee (IACUC2016-0098), and animal care and use complied with all relevant local animal welfare laws, guidelines and policies. Mice were housed in a vivarium with a 12 h-12 h light-dark cycle, with food and water *ad libitum*.

### Histological analysis

Neonatal pups were sacrificed by decapitation. For histology of the adults, littermate animals underwent cardiac perfusion using cold heparinized PBS and formalin (Sigma-Aldrich) solution. Brains, lungs and testes were dissected and fixed for ≤72 h in formalin at room temperature, followed by immersion in 70% ethanol (for histology). Samples were then paraffin embedded and sectioned at 6 µm thickness. Sections were processed for Hematoxylin and Eosin staining with standard methods. Histological sections were imaged using a Zeiss Discovery.V8 Stereoscope or Nikon NIE upright microscope. The area of the lateral ventricles was measured from the coronal brain sections. We limited our analysis to sections that contained the anterior commissure as an anatomical landmark for consistency between wild-type and *Spag17^Pcdo/Pcdo^* mutant animals. Analysis was performed using the area measurement tool within the Nikon Element 4.50 software.

### Immunohistochemistry

Immunohistochemistry was performed as previously described by Driver et al. (2017). Briefly, fixed paraffin blocks from control and mutant animals were sectioned at a thickness of 6 µm. Sections were deparaffinized and rehydrated in graded ethanol. Antigen retrieval was performed by boiling in citrate buffer pH 6.00 for 1 min in a microwave. Nonspecific antigens in the tissue sections were blocked in 4% normal goat serum in PBS-Tween, and the anti-acetylated α-tubulin antibody (1:2000, Sigma-Aldrich, cat# T6793) was incubated with the tissue overnight at 4°C, followed by three stringent washes in PBS-Tween and application of Alexa Fluor 596-conjugated goat anti-mouse secondary antibody (1:500, Invitrogen, cat# A11005) for 1 h, followed by three washes in PBS-Tween. Nuclei were counterstained with 4′,6-diamidino-2-phenylindole (DAPI) and mounted in ProLong Gold antifade mounting media (Invitrogen). Images were captured using a Nikon C2 confocal microscope.

### Whole-exome sequencing

Brain tissue from three obviously affected, postnatal male mice was used for exome analysis. Whole-exome sequencing was done with BGI Americas, using standard protocols. Annotation of single nucleotide polymorphisms was performed at BGI Americas with an in-house software package, ‘AnnoDB’, incorporating elements from analysis platforms such as Sorting Intolerant From Tolerant (SIFT), Polymorphism Phenotyping v.2 (PolyPhen-2), phylogenetic *P*-values (PhyloP), GERP (Genome Evolutionary Rate Profiling) and Functional Analysis through Hidden Markov Models (FATHMM).

### Sanger sequencing

The *Spag17* nonsense variant in exon 36, identified by whole-exome sequencing, was confirmed by Sanger sequencing using the following primers: forward, 5′-TTTGCCATTTGGATTTACAGG-3′; and reverse, 5′-GTAGCACTTTGGGTCCTTCG-3′. PCR amplicons from *Spag17^wt/wt^*, *Spag17^Pcdo/wt^* and *Spag17^Pcdo/Pcdo^* animals were purified and concentrated using the Zymo DNA Clean and Concentrator kit (Zymo Research Corporation). One hundred nanograms of concentrated DNA was used in each Sanger sequencing reaction.

### *Spag17^Pcdo^* mouse line genotyping assays

All animals and pups obtained from the *Spag17^Pcdo^* mouse line were genotyped using the TaqMan Sample-to-SNP kit (Applied Biosystems) for a single-nucleotide change at mouse chr1:100088282A>T (assay ID ANWCWCA). *Spag17^Pcdo^* TaqMan Sample-to-SNP assays were performed according to the manufacturer's instructions and run using a QuantStudio 6 Real Time PCR machine (Applied Biosystems).

### Semi-quantitative PCR (RT-PCR)

Total RNA was extracted from testes, lungs and whole brains of adult mice using TRIzol reagent (Thermo Fisher Scientific) according to the manufacturer's instructions. Five micrograms of total RNA was used for reverse transcription into complementary DNA (cDNA) using the SuperScript III First Strand synthesis system (Invitrogen) according to the manufacturer's directions. Final cDNA products were diluted 1:10, after which 2 µl of the diluted cDNA was used to amplify *Spag17* transcript with primers that span exon 33 and exon 39 (forward, 5′-GATGGAGGGCTACGAAAGC-3′; reverse, 5′-AACTGTTAGGTGGGCTGCAA-3′). β-Actin was used as loading control (forward, 5′-GTGACGTTGACATCCGTAAAGA-3′; reverse, 5′-GCCGGACTCATCGTACTCC-3′). PCR products were run on a 2% agarose gel with ethidium bromide for 45 min at 120 V. PCR bands were imaged using a Bio-Rad Universal Hood II Molecular Imager w/CFW-1312M Camera equipped with Image Lab v.6.0.1 software. The image analysis tool in the Image Lab v.6.0.1 software was used to quantify the band intensity value. Data obtained from the *Spag17* exon 33-39 bands were normalized against the β-actin value of the corresponding sample. Finally, wild-type and mutant data obtained for testes, lungs and brain were normalized against the average wild-type testis expression values. One-way ANOVA with multiple comparisons statistical analyses were performed using GraphPad Prism v.8.0.1 software.

### Western immunoblotting

Adult mouse brain and testis tissues were lysed in Pierce RIPA buffer (Thermo Fisher Scientific, cat# 89901) containing protease inhibitor cocktail (Roche, cat# 11697498001). The protein concentration in the whole cell extracts was determined with the BCA colorimetric assay (Thermo Fisher Scientific) according to the manufacturer's instructions. Denatured proteins were separated by electrophoresis on a gradient of 4-12% Tris-glycine gel. The protein was transferred to a polyvinylidene difluoride membrane, blocked in Odyssey blocking buffer and incubated overnight at 4°C with 1:3000 rabbit anti-N-terminus or C-terminus anti-SPAG17 antibodies (Zhang et al., 2005) and 1:2000 mouse anti-tubulin antibodies (Sigma-Aldrich, cat# T6199). Membranes were washed and incubated for 1 h in 1:15,000 goat anti-rabbit IRDye 680CW (LICOR) and 1:15,000 goat anti-mouse IRDye 800Rd (LICOR), and bands were visualized on the LICOR Odyssey imaging system.

### Scanning electron microscopy

The medial and lateral walls of the P8 forebrains were processed for SEM as previously described (Abdelhamed et al., 2018). Briefly, samples were fixed in electron microscopy grade 2% paraformaldehyde and 2.5% glutaraldehyde in 0.1 M sodium cacodylate buffer (pH 7.4) at 4°C overnight. Tissues were washed thoroughly in 0.1 M sodium cacodylate buffer (pH 7.4) then post-fixed in 1% osmium oxide (diluted in 0.1 M sodium cacodylate buffer) for 1 h. Samples were washed thoroughly in 0.1 M sodium cacodylate buffer and dehydrated before critical point drying in 100% ethanol. Brain tissues were then coated with gold palladium using a sputter coater (Leica EM ACE600) and scanned with a Hitachi SU8O1O scanning electron microscope.

### TEM

Brain ependymal cells and tracheal epithelial cells were fixed in 2.5% glutaraldehyde and processed for TEM analyses by standard protocols as previously reported (Wallmeier et al., 2019). Sections were collected on copper grids, stained with Reynold's lead citrate and visualized using the Philips CM10 or Jeol 1400+.

### High-speed video microscopy of cilia, ciliary beat frequency and CSF flow analysis

High-speed video microscopy of the beating ependymal cilia, ciliary beat frequency and CSF flow analysis were performed as previously described (Abdelhamed et al., 2018). Briefly, brains were dissected out from P4 *Spag17^Pcdo/Pcdo^* or wild-type control animals in Dulbecco's modified Eagle's medium: Nutrient Mixture F-12 (DMEM/F12) supplemented with l-glutamine (Gibco) and 1% N2 supplement (Gibco) at room temperature, and cut into serial 200-µm-thick coronal sections. Sections were obtained from the forebrain and aqueduct. Areas from the medial wall of the lateral ventricle were microdissected further and subjected to video microscopy recording at ∼100 frames/s. These videos were used to measure the ciliary beat frequency. For concurrent imaging of the beating cilia with green fluorescent microbeads (FluoSpheres, Thermo Fisher Scientific), the beads were introduced into slices immediately before video recording, and we used an inverted Nikon Ti-E wide-field microscope fitted with a Nikon ×40 Plan Apo 0.95 N.A. air objective and an Andor Zyla 4.2 PLUS sCMOS monochromatic camera. Light was channeled through a custom quad-pass filter, and 300 frames were collected at ∼60 frames/s. Bead tracking and ciliary beat frequency analysis were performed using NIS Element software v.4.5. Statistical analysis and pairwise comparisons were performed using GraphPad Prism software.

### Skeletal preparations

For skeletal preparations, adult animals were sacrificed by induction of deep anesthesia using isoflurane, followed by trans-diaphragmatic cardiac extrusion. Animals were eviscerated and fixed for 2 days in 95% ethanol. They were stained overnight at room temperature in Alcian Blue solution (Sigma-Aldrich, cat# A3157) containing 20% glacial acetic acid. Excess stain was cleared in 95% ethanol for 24 h, and skeletons were then slightly cleared in a 1% KOH solution overnight at room temperature. They were then stained overnight in Alizarin Red solution (Sigma-Aldrich, cat# A5533) containing 1% KOH. A second round of clearing was performed by incubating tissues in 20% glycerol/1% KOH solution for 24 h. Finally, they were transferred to 50% glycerol/50% ethanol for photography. Skeletal preparations were imaged using a Zeiss Discovery.V8 Stereoscope.

### Generation, culture and immunocytochemistry of MEFs

MEFs were generated from embryonic day (E)13.5 embryos. Embryos were dissected in PBS, decapitated and eviscerated. The remaining tissue was incubated in trypsin overnight at 4°C. Tissue fragments were incubated with the trypsin in a 5% CO_2_ incubator for 30 min. Cells were then allowed to grow to confluency in complete DMEM containing 10% fetal bovine serum and penicillin/streptomycin. MEFs were stained within three passages of their isolation. Cells were stained for the ciliary membrane marker ARL13B (1:500, ProteinTech, cat# 17711-1-AP) overnight at 4°C, after blocking nonspecific antigenic sites with 4% normal goat serum in PBS-Tween for 1 h. Alexa Fluor 488-conjugated goat anti-rabbit secondary antibody (1/500, Invitrogen, cat# A11008) for 1 h, and nuclei were stained with DAPI.

### Reagents, methodology and statistical analysis

MEFs were generated in the laboratory directly from mouse embryos with standard protocols. All antibodies (with the exception of anti-SPAG17) used in this study were commercially available, and our results matched multiple published reports. SPAG17 results were consistent with previous results, and we saw loss of SPAG17 immunoblot signals in *Spag17* mutants, which is ideal validation of an antibody. Sample sizes were generally selected by historical precedent in the field of three or more mutant/control pairs from three different litters, as far as possible (see Discussion for study limitations for some sample sizes). This assignment of mutant/control from different litters also accounted for allocation to experimental groups. Most of the analyses presented in this article were performed by an investigator blinded to the genotypes until after initial data collection. This was possible because the initial tissue collections and genotyping PCRs were often done by a different member of the scientific team. Samples or animals were excluded from the study only in the case of pre-established and clear experimental failures; these failures included immunohistochemistry, immunocytochemistry or immunoblots where no appropriate signal was obtained for any specimens in the experiment. No animals in obvious distress or showing signs of morbidity were used in this study, with the exception of the observations of intraventricular hemorrhage presented in the supplemental data. GraphPad Prism v.8.0.1 software (GraphPad Software, LLC) was used to perform all statistical analysis presented in this study. Analysis also included an analysis for appropriate methodology and sample distribution(s) for the appropriate test. Paired comparisons were made with Student's *t*-test (two tailed), and multiple sample data sets were analyzed with an ANOVA followed by multiple comparison testing. Graphs all use lines to show the mean±s.d.

## Supplementary Material

Supplementary information

## References

[DMM045344C1] Abdelhamed Z., Vuong S. M., Hill L., Shula C., Timms A., Beier D., Campbell K., Mangano F. T., Stottmann R. W. and Goto J. (2018). A mutation in Ccdc39 causes neonatal hydrocephalus with abnormal motile cilia development in mice. *Development* 145, dev154500 10.1242/dev.154500 29317443 PMC5825874

[DMM045344C2] Adams G. M., Huang B., Piperno G. and Luck D. J. (1981). Central-pair microtubular complex of Chlamydomonas flagella: polypeptide composition as revealed by analysis of mutants. *J. Cell Biol.* 91, 69-76. 10.1083/jcb.91.1.69 7028763 PMC2111942

[DMM045344C4] Afzelius B. A. (1981). Genetical and ultrastructural aspects of the immotile-cilia syndrome. *Am. J. Hum. Genet.* 33, 852-864. 7034533 PMC1685161

[DMM045344C5] Andjelkovic M., Minic P., Vreca M., Stojiljkovic M., Skakic A., Sovtic A., Rodic M., Skodric-Trifunovic V., Maric N., Visekruna J. et al. (2018). Genomic profiling supports the diagnosis of primary ciliary dyskinesia and reveals novel candidate genes and genetic variants. *PLoS ONE* 13, e0205422-e0205422. 10.1371/journal.pone.0205422 30300419 PMC6177184

[DMM045344C6] Bartoloni L., Blouin J.-L., Pan Y., Gehrig C., Maiti A. K., Scamuffa N., Rossier C., Jorissen M., Armengot M., Meeks M. et al. (2002). Mutations in the DNAH11 (axonemal heavy chain dynein type 11) gene cause one form of situs inversus totalis and most likely primary ciliary dyskinesia. *Proc. Natl. Acad. Sci. USA* 99, 10282-10286. 10.1073/pnas.152337699 12142464 PMC124905

[DMM045344C7] Berlucchi M., de Santi M. M., Bertoni E., Spinelli E., Timpano S. and Padoan R. (2012). Ciliary aplasia associated with hydrocephalus: an extremely rare occurrence. *Eur. Arch. Oto-Rhino-Laryn.* 269, 2295-2299. 10.1007/s00405-012-2107-3 22791471

[DMM045344C8] Blackshear P. J., Graves J. P., Stumpo D. J., Cobos I., Rubenstein J. L. R. and Zeldin D. C. (2003). Graded phenotypic response to partial and complete deficiency of a brain-specific transcript variant of the winged helix transcription factor RFX4. *Development* 130, 4539 10.1242/dev.00661 12925582

[DMM045344C9] Boisvieux-Ulrich E., Laine M. C. and Sandoz D. (1985). The orientation of ciliary basal bodies in quail oviduct is related to the ciliary beating cycle commencement. *Biol. Cell* 55, 147-150. 10.1111/j.1768-322X.1985.tb00417.x 2937490

[DMM045344C10] Brightman M. W. and Palay S. L. (1963). The fine structure of ependyma in the brain of the rat. *J. Cell Biol.* 19, 415-439. 10.1083/jcb.19.2.415 14086765 PMC2106872

[DMM045344C11] Brody S. L., Yan X. H., Wuerffel M. K., Song S.-K. and Shapiro S. D. (2000). Ciliogenesis and left–right axis defects in forkhead factor HFH-4–null mice. *Am. J. Respir. Cell Mol. Biol.* 23, 45-51. 10.1165/ajrcmb.23.1.4070 10873152

[DMM045344C12] Brueckner M. (2001). Cilia propel the embryo in the right direction. *Am. J. Med. Genet.* 101, 339-344. 10.1002/1096-8628(20010715)101:4<339::AID-AJMG1442>3.0.CO;2-P 11471157

[DMM045344C13] Bukowy-Bieryłło Z., Ziętkiewicz E., Loges N. T., Wittmer M., Geremek M., Olbrich H., Fliegauf M., Voelkel K., Rutkiewicz E., Rutland J. et al. (2013). RPGR mutations might cause reduced orientation of respiratory cilia. *Pediatr. Pulmanol.* 48, 352-363. 10.1002/ppul.22632 22888088

[DMM045344C14] Bustamante-Marin X. M., Yin W.-N., Sears P. R., Werner M. E., Brotslaw E. J., Mitchell B. J., Jania C. M., Zeman K. L., Rogers T. D., Herring L. E. et al. (2019). Lack of GAS2L2 causes PCD by impairing cilia orientation and mucociliary clearance. *Am. J. Hum. Genet.* 104, 229-245. 10.1016/j.ajhg.2018.12.009 30665704 PMC6372263

[DMM045344C15] Cao M. and Wu J. I. (2015). Camk2a-Cre-mediated conditional deletion of chromatin remodeler Brg1 causes perinatal hydrocephalus. *Neurosci. Lett.* 597, 71-76. 10.1016/j.neulet.2015.04.041 25929186 PMC4444043

[DMM045344C16] Cindrić S., Dougherty G. W., Olbrich H., Hjeij R., Loges N. T., Amirav I., Philipsen M. C., Marthin J. K., Nielsen K. G., Sutharsan S. et al. (2020). SPEF2- and HYDIN-mutant cilia lack the central pair associated protein SPEF2 aiding primary ciliary dyskinsea diagnostics. *Am. J. Respir. Cell Mol. Biol.* 62, 382-396. 10.1165/rcmb.2019-0086OC 31545650

[DMM045344C17] Córdova-Fletes C., Becerra-Solano L. E., Rangel-Sosa M. M., Rivas-Estilla A. M., Alberto Galán-Huerta K., Ortiz-López R., Rojas-Martínez A., Juárez-Vázquez C. I. and García-Ortiz J. E. (2018). Uncommon runs of homozygosity disclose homozygous missense mutations in two ciliopathy-related genes (SPAG17 and WDR35) in a patient with multiple brain and skeletal anomalies. *Eur. J. Med. Genet.* 61, 161-167. 10.1016/j.ejmg.2017.11.011 29174089

[DMM045344C18] Davy B. E. and Robinson M. L. (2003). Congenital hydrocephalus in hy3 mice is caused by a frameshift mutation in Hydin, a large novel gene. *Hum. Mol. Genet.* 12, 1163-1170. 10.1093/hmg/ddg122 12719380

[DMM045344C19] Del Bigio M. R. (2010). Ependymal cells: biology and pathology. *Acta Neuropathol.*. 119, 55-73. 10.1007/s00401-009-0624-y 20024659

[DMM045344C20] Dirksen E. R. (1971). Centriole morphogenesis in developing ciliated epithelium of the mouse oviduct. *J. Cell Biol.* 51, 286-302. 10.1083/jcb.51.1.286 5111878 PMC2108250

[DMM045344C21] Dougherty G. W., Loges N. T., Klinkenbusch J. A., Olbrich H., Pennekamp P., Menchen T., Raidt J., Wallmeier J., Werner C., Westermann C. et al. (2016). DNAH11 localization in the proximal region of respiratory cilia defines distinct outer dynein arm complexes. *Am. J. Respir. Cell Mol. Biol.* 55, 213-224. 10.1165/rcmb.2015-0353OC 26909801 PMC4979367

[DMM045344C22] Driver A. M., Shumrick C. and Stottmann R. W. (2017). Ttc21b is required in bergmann glia for proper granule cell radial migration. *J. Dev. Biol.* 5, 18 10.3390/jdb5040018 PMC5831799 29615573

[DMM045344C23] Dutcher S. K., Huang B. and Luck D. J. (1984). Genetic dissection of the central pair microtubules of the flagella of Chlamydomonas reinhardtii. *J. Cell Biol.* 98, 229-236. 10.1083/jcb.98.1.229 6707088 PMC2113000

[DMM045344C24] Edelbusch C., Cindrić S., Dougherty G. W., Loges N. T., Olbrich H., Rivlin J., Wallmeier J., Pennekamp P., Amirav I. and Omran H. (2017). Mutation of serine/threonine protein kinase 36 (STK36) causes primary ciliary dyskinesia with a central pair defect. *Hum. Mutat.* 38, 964-969. 10.1002/humu.23261 28543983

[DMM045344C25] Ferkol T. W. and Leigh M. W. (2012). Ciliopathies: the central role of cilia in a spectrum of pediatric disorders. *J. Pediatr.* 160, 366-371. 10.1016/j.jpeds.2011.11.024 22177992 PMC3282141

[DMM045344C26] Fernandez-Gonzalez A., Kourembanas S., Wyatt T. A. and Mitsialis S. A. (2009). Mutation of murine adenylate kinase 7 underlies a primary ciliary dyskinesia phenotype. *Am. J. Respir. Cell Mol. Biol.* 40, 305-313. 10.1165/rcmb.2008-0102OC 18776131 PMC2645528

[DMM045344C27] Finn R., Evans C. C. and Lee L. (2014). Strain-dependent brain defects in mouse models of primary ciliary dyskinesia with mutations in Pcdp1 and Spef2. *Neuroscience* 277, 552-567. 10.1016/j.neuroscience.2014.07.029 25073043 PMC4164581

[DMM045344C28] Fliegauf M., Benzing T. and Omran H. (2007). When cilia go bad: cilia defects and ciliopathies. *Nat. Rev. Mol. Cell Biol.* 8, 880-893. 10.1038/nrm2278 17955020

[DMM045344C29] Fu G., Zhao L., Dymek E., Hou Y., Song K., Phan N., Shang Z., Smith E. F., Witman G. B. and Nicastro D. (2019). Structural organization of the C1a-e-c supercomplex within the ciliary central apparatus. *J. Cell Biol.* 218, 4236-4251. 10.1083/jcb.201906006 31672705 PMC6891083

[DMM045344C30] Gibbons I. R. (1961). The relationship between the fine structure and direction of beat in gill cilia of a lamellibranch mollusc. *J. Biophys. Biochem. Cytol.* 11, 179-205. 10.1083/jcb.11.1.179 13898346 PMC2225111

[DMM045344C31] Gilula N. B. and Satir P. (1972). The ciliary necklace: a ciliary membrane specialization. *J. Cell Biol.* 53, 494-509. 10.1083/jcb.53.2.494 4554367 PMC2108734

[DMM045344C32] Goduti D. J. and Smith E. F. (2012). Analyses of functional domains within the PF6 protein of the central apparatus reveal a role for PF6 sub-complex members in regulating flagellar beat frequency. *Cytoskeleton* 69, 179-194. 10.1002/cm.21010 22278927 PMC3309106

[DMM045344C33] Goetz S. C. and Anderson K. V. (2010). The primary cilium: a signalling centre during vertebrate development. *Nat. Rev. Genet.* 11, 331-344. 10.1038/nrg2774 20395968 PMC3121168

[DMM045344C34] Gokce O., Stanley G. M., Treutlein B., Neff N. F., Camp J. G., Malenka R. C., Rothwell P. E., Fuccillo M. V., Südhof T. C. and Quake S. R. (2016). Cellular taxonomy of the mouse striatum as revealed by single-cell RNA-seq. *Cell Rep.* 16, 1126-1137. 10.1016/j.celrep.2016.06.059 27425622 PMC5004635

[DMM045344C35] Ha S., Lindsay A. M., Timms A. E. and Beier D. R. (2016). Mutations in Dnaaf1 and Lrrc48 cause hydrocephalus, laterality defects, and sinusitis in mice. *G3 (Bethesda)* 6, 2479-2487. 10.1534/g3.116.030791 27261005 PMC4978901

[DMM045344C36] Handel M. A. and Kennedy J. R. (1984). Situs inversus in homozygous mice without immotile cilia. *J. Hered.* 75, 498-498. 10.1093/oxfordjournals.jhered.a109995 6512242

[DMM045344C37] Hegan P. S., Ostertag E., Geurts A. M. and Mooseker M. S. (2015). Myosin Id is required for planar cell polarity in ciliated tracheal and ependymal epithelial cells. *Cytoskeleton* 72, 503-516. 10.1002/cm.21259 26446290 PMC4715625

[DMM045344C38] Herron B. J., Lu W., Rao C., Liu S., Peters H., Bronson R. T., Justice M. J., McDonald J. D. and Beier D. R. (2002). Efficient generation and mapping of recessive developmental mutations using ENU mutagenesis. *Nat. Genet.* 30, 185-189. 10.1038/ng812 11818962

[DMM045344C39] Huang B., Ramanis Z. and Luck D. J. L. (1982). Suppressor mutations in chlamydomonas reveal a regulatory mechanism for flagellar function. *Cell* 28, 115-124. 10.1016/0092-8674(82)90381-6 6461414

[DMM045344C40] Ibañez-Tallon I., Pagenstecher A., Fliegauf M., Olbrich H., Kispert A., Ketelsen U.-P., North A., Heintz N. and Omran H. (2004). Dysfunction of axonemal dynein heavy chain Mdnah5 inhibits ependymal flow and reveals a novel mechanism for hydrocephalus formation. *Hum. Mol. Genet.* 13, 2133-2141. 10.1093/hmg/ddh219 15269178

[DMM045344C41] Inaba K. (2011). Sperm flagella: comparative and phylogenetic perspectives of protein components. *MHR Basic Sci. Reprod. Med.* 17, 524-538. 10.1093/molehr/gar034 21586547

[DMM045344C42] Jeffery P. K. and Reid L. (1975). New observations of rat airway epithelium: a quantitative and electron microscopic study. *J. Anat.* 120, 295-320. 1201964 PMC1231972

[DMM045344C43] Kazarian E., Son H. Y., Sapao P., Li W., Zhang Z., Strauss J. F. and Teves M. E. (2018). SPAG17 is required for male germ cell differentiation and fertility. *Int. J. Mol. Sci.* 19, 1252 10.3390/ijms19041252 PMC5979577 29690537

[DMM045344C44] Kim J.-J., Lee H.-I., Park T., Kim K., Lee J.-E., Cho N. H., Shin C., Cho Y. S., Lee J.-Y., Han B.-G. et al. (2010). Identification of 15 loci influencing height in a Korean population. *J. Hum. Genet.* 55, 27-31. 10.1038/jhg.2009.116 19893584

[DMM045344C45] Knowles M. R., Leigh M. W., Carson J. L., Davis S. D., Dell S. D., Ferkol T. W., Olivier K. N., Sagel S. D., Rosenfeld M., Burns K. A. et al. (2012). Mutations of DNAH11 in patients with primary ciliary dyskinesia with normal ciliary ultrastructure. *Thorax* 67, 433-441. 10.1136/thoraxjnl-2011-200301 22184204 PMC3739700

[DMM045344C46] Kosaki K., Ikeda K., Miyakoshi K., Ueno M., Kosaki R., Takahashi D., Tanaka M., Torikata C., Yoshimura Y. and Takahashi T. (2004). Absent inner dynein arms in a fetus with familial hydrocephalus-situs abnormality. *Am. J. Med. Genet.* 129A, 308-311. 10.1002/ajmg.a.30177 15326634

[DMM045344C47] Kunimoto K., Yamazaki Y., Nishida T., Shinohara K., Ishikawa H., Hasegawa T., Okanoue T., Hamada H., Noda T., Tamura A. et al. (2012). Coordinated ciliary beating requires Odf2-mediated polarization of basal bodies via basal feet. *Cell* 148, 189-200. 10.1016/j.cell.2011.10.052 22265411

[DMM045344C48] Kurkowiak M., Ziętkiewicz E. and Witt M. (2015). Recent advances in primary ciliary dyskinesia genetics. *J. Med. Genet.* 52, 1 10.1136/jmedgenet-2014-102755 25351953 PMC4285891

[DMM045344C49] Lechtreck K.-F., Delmotte P., Robinson M. L., Sanderson M. J. and Witman G. B. (2008). Mutations in Hydin impair ciliary motility in mice. *J. Cell Biol.* 180, 633 10.1083/jcb.200710162 18250199 PMC2234243

[DMM045344C50] Lee L. (2011). Mechanisms of mammalian ciliary motility: Insights from primary ciliary dyskinesia genetics. *Gene* 473, 57-66. 10.1016/j.gene.2010.11.006 21111794

[DMM045344C51] Lee L. (2013). Riding the wave of ependymal cilia: Genetic susceptibility to hydrocephalus in primary ciliary dyskinesia. *J. Neurosci. Res.* 91, 1117-1132. 10.1002/jnr.23238 23686703

[DMM045344C52] Lee L., Campagna D. R., Pinkus J. L., Mulhern H., Wyatt T. A., Sisson J. H., Pavlik J. A., Pinkus G. S. and Fleming M. D. (2008). Primary ciliary dyskinesia in mice lacking the novel ciliary protein Pcdp1. *Mol. Cell. Biol.* 28, 949-957. 10.1128/MCB.00354-07 18039845 PMC2223405

[DMM045344C53] Leigh M. W., Horani A., Kinghorn B., O'Connor M. G., Zariwala M. A. and Knowles M. R. (2019). Primary Ciliary Dyskinesia (PCD): a genetic disorder of motile cilia. *Transl. Sci. Rare Dis.* 4, 51-75. 10.3233/TRD-190036 31572664 PMC6768089

[DMM045344C54] Loges N. T., Antony D., Maver A., Deardorff M. A., Güleç E. Y., Gezdirici A., Nöthe-Menchen T., Höben I. M., Jelten L., Frank D. et al. (2018). Recessive DNAH9 loss-of-function mutations cause laterality defects and subtle respiratory ciliary-beating defects. *Am. J. Hum. Genet.* 103, 995-1008. 10.1016/j.ajhg.2018.10.020 30471718 PMC6288205

[DMM045344C55] Lorès P., Coutton C., El Khouri E., Stouvenel L., Givelet M., Thomas L., Rode B., Schmitt A., Louis B., Sakheli Z. et al. (2018). Homozygous missense mutation L673P in adenylate kinase 7 (AK7) leads to primary male infertility and multiple morphological anomalies of the flagella but not to primary ciliary dyskinesia. *Hum. Mol. Genet.* 27, 1196-1211. 10.1093/hmg/ddy034 29365104

[DMM045344C56] Lucas J. S., Davis S. D., Omran H. and Shoemark A. (2019). Primary ciliary dyskinesia in the genomics age. *Lancet Respir. Med.* 8, 202-216. 10.1016/S2213-2600(19)30374-1 31624012

[DMM045344C57] Marszalek J. R., Ruiz-Lozano P., Roberts E., Chien K. R. and Goldstein L. S. B. (1999). Situs inversus and embryonic ciliary morphogenesis defects in mouse mutants lacking the KIF3A subunit of kinesin-II. *Proc. Natl Acad. Sci. USA* 96, 5043 10.1073/pnas.96.9.5043 10220415 PMC21813

[DMM045344C58] Mitchell M. J., Woods D. R., Tucker P. K., Opp J. S. and Bishop C. E. (1991). Homology of a candidate spermatogenic gene from the mouse Y chromosome to the ubiquitin-activating enzyme El. *Nature* 354, 483-486. 10.1038/354483a0 1684224

[DMM045344C59] Mitchell B., Jacobs R., Li J., Chien S. and Kintner C. (2007). A positive feedback mechanism governs the polarity and motion of motile cilia. *Nature* 447, 97-101. 10.1038/nature05771 17450123

[DMM045344C60] Mochida K., Tres L. L. and Kierszenbaum A. L. (2000). Structural features of the 26S proteasome complex isolated from rat testis and sperm tail. *Mol. Reprod. Dev.* 57, 176-184. 10.1002/1098-2795(200010)57:2<176::AID-MRD9>3.0.CO;2-O 10984418

[DMM045344C61] Moore A., Escudier E., Roger G., Tamalet A., Pelosse B., Marlin S., Clément A., Geremek M., Delaisi B., Bridoux A.-M. et al. (2006). RPGR is mutated in patients with a complex X linked phenotype combining primary ciliary dyskinesia and retinitis pigmentosa. *J. Med. Genet.* 43, 326-333. 10.1136/jmg.2005.034868 16055928 PMC2563225

[DMM045344C62] Morales P., Kong M., Pizarro E. and Pasten C. (2003). Participation of the sperm proteasome in human fertilization. *Hum. Reprod.* 18, 1010-1017. 10.1093/humrep/deg111 12721178

[DMM045344C63] N'Diaye A., Chen G. K., Palmer C. D., Ge B., Tayo B., Mathias R. A., Ding J., Nalls M. A., Adeyemo A., Adoue V. et al. (2011). Identification, replication, and fine-mapping of loci associated with adult height in individuals of African ancestry. *PLoS Genet.* 7, e1002298 10.1371/journal.pgen.1002298 21998595 PMC3188544

[DMM045344C64] Nishito Y., Hasegawa M., Inohara N. and Núñez G. (2006). MEX is a testis-specific E3 ubiquitin ligase that promotes death receptor-induced apoptosis. *Biochem. J.* 396, 411-417. 10.1042/BJ20051814 16522193 PMC1482824

[DMM045344C65] Nogales E., Whittaker M., Milligan R. A. and Downing K. H. (1999). High-resolution model of the microtubule. *Cell* 96, 79-88. 10.1016/S0092-8674(00)80961-7 9989499

[DMM045344C66] Nonaka S., Tanaka Y., Okada Y., Takeda S., Harada A., Kanai Y., Kido M. and Hirokawa N. (1998). Randomization of left–right asymmetry due to loss of Nodal Cilia generating leftward flow of extraembryonic fluid in mice lacking KIF3B motor protein. *Cell* 95, 829-837. 10.1016/S0092-8674(00)81705-5 9865700

[DMM045344C67] Pasterkamp R. J., Peschon J. J., Spriggs M. K. and Kolodkin A. L. (2003). Semaphorin 7A promotes axon outgrowth through integrins and MAPKs. *Nature* 424, 398-405. 10.1038/nature01790 12879062

[DMM045344C68] Pazour G. J., Dickert B. L., Vucica Y., Seeley E. S., Rosenbaum J. L., Witman G. B. and Cole D. G. (2000). Chlamydomonas IFT88 and its mouse homologue, polycystic kidney disease gene Tg737, are required for assembly of cilia and flagella. *J. Cell Biol.* 151, 709-718. 10.1083/jcb.151.3.709 11062270 PMC2185580

[DMM045344C69] Pedersen L. B., Veland I. R., Schrøder J. M. and Christensen S. T. (2008). Assembly of primary cilia. *Dev. Dyn.* 237, 1993-2006. 10.1002/dvdy.21521 18393310

[DMM045344C70] Pérez-Fígares J. M., Jimenez A. J. and Rodríguez E. M. (2001). Subcommissural organ, cerebrospinal fluid circulation, and hydrocephalus. *Microsc. Res. Tech.* 52, 591-607. 10.1002/1097-0029(20010301)52:5<591::AID-JEMT1043>3.0.CO;2-7 11241868

[DMM045344C71] Pifferi M., Michelucci A., Conidi M. E., Cangiotti A. M., Simi P., Macchia P. and Boner A. L. (2010). New DNAH11 mutations in primary ciliary dyskinesia with normal axonemal ultrastructure. *Eur. Res. J.* 35, 1413-1416. 10.1183/09031936.00186209 20513915

[DMM045344C72] Porter M. E., Power J. and Dutcher S. K. (1992). Extragenic suppressors of paralyzed flagellar mutations in Chlamydomonas reinhardtii identify loci that alter the inner dynein arms. *J. Cell Biol.* 118, 1163 10.1083/jcb.118.5.1163 1387404 PMC2289579

[DMM045344C73] Porter M. E., Knott J. A., Gardner L. C., Mitchell D. R. and Dutcher S. K. (1994). Mutations in the SUP-PF-1 locus of Chlamydomonas reinhardtii identify a regulatory domain in the beta-dynein heavy chain. *J. Cell Biol.* 126, 1495 10.1083/jcb.126.6.1495 8089181 PMC2290962

[DMM045344C74] Rodríguez E. M., Hein S., Rodríguez S., Herrera H., Peruzzo B., Nualart F. and Oksche A. (1987). Analysis of the secretory products of the subcommissural organ. In *Functional Morphology of Neuroendocrine Systems: Evolutionary and Environmental Aspects* (ed. Scharrer B., Korf H.-W. and Hartwig H.-G.), pp. 189-202. Berlin, Heidelberg: Springer Berlin Heidelberg.

[DMM045344C75] Rupp G., O'Toole E., Gardner L. C., Mitchell B. F. and Porter M. E. (1996). The sup-pf-2 mutations of Chlamydomonas alter the activity of the outer dynein arms by modification of the gamma-dynein heavy chain. *J. Cell Biol.* 135, 1853 10.1083/jcb.135.6.1853 8991096 PMC2133962

[DMM045344C76] Rupp G., O'Toole E. and Porter M. E. (2001). The Chlamydomonas PF6 locus encodes a large alanine/proline-rich polypeptide that is required for assembly of a central pair projection and regulates flagellar motility. *Mol. Biol. Cell* 12, 739-751. 10.1091/mbc.12.3.739 11251084 PMC30977

[DMM045344C77] Sakakibara S.-I., Nakamura Y., Yoshida T., Shibata S., Koike M., Takano H., Ueda S., Uchiyama Y., Noda T. and Okano H. (2002). RNA-binding protein Musashi family: roles for CNS stem cells and a subpopulation of ependymal cells revealed by targeted disruption and antisense ablation. *Proc. Natl. Acad. Sci. USA* 99, 15194-15199. 10.1073/pnas.232087499 12407178 PMC137566

[DMM045344C78] Sánchez I. and Dynlacht B. D. (2016). Cilium assembly and disassembly. *Nat. Cell Biol.* 18, 711-717. 10.1038/ncb3370 27350441 PMC5079433

[DMM045344C79] Satir P. (2005). Tour of organelles through the electron microscope: a reprinting of Keith R. Porter's classic Harvey Lecture with a new introduction. *Anat. Rec. A Discov. Mol. Cell. Evol. Biol.* 287A, 1184-1204. 10.1002/ar.a.20222 16265625

[DMM045344C80] Satir P. and Sleigh M. A. (1990). The physiology of cilia and mucociliary interactions. *Annu. Rev. Physiol.* 52, 137-155. 10.1146/annurev.ph.52.030190.001033 2184754

[DMM045344C81] Schwabe G. C., Hoffmann K., Loges N. T., Birker D., Rossier C., de Santi M. M., Olbrich H., Fliegauf M., Failly M., Liebers U. et al. (2008). Primary ciliary dyskinesia associated with normal axoneme ultrastructure is caused by DNAH11 mutations. *Hum. Mutant.* 29, 289-298. 10.1002/humu.20656 18022865

[DMM045344C82] Silina K., Zayakin P., Kalnina Z., Ivanova L., Meistere I., Endzelinš E., Abols A., Stengrevics A., Leja M., Ducena K. et al. (2011). Sperm-associated antigens as targets for cancer immunotherapy: expression pattern and humoral immune response in cancer patients. *J. Immunother.* 34, 28-44. 10.1097/CJI.0b013e3181fb64fa 21150711

[DMM045344C83] Sironen A., Kotaja N., Mulhern H., Wyatt T. A., Sisson J. H., Pavlik J. A., Miiluniemi M., Fleming M. D. and Lee L. (2011). Loss of SPEF2 function in mice results in spermatogenesis defects and primary ciliary Dyskinesia1. *Biol. Reprod.* 85, 690-701. 10.1095/biolreprod.111.091132 21715716 PMC3184289

[DMM045344C84] Smith E. F. and Yang P. (2004). The radial spokes and central apparatus: mechano-chemical transducers that regulate flagellar motility. *Cell Motil. Cytoskelet.* 57, 8-17. 10.1002/cm.10155 PMC1950942 14648553

[DMM045344C85] Stottmann R. and Beier D. (2014). ENU mutagenesis in the mouse. *Curr. Protoc.* 4, 25-35. 10.1002/9780470942390.mo140029 25723916

[DMM045344C86] Stoykova A., Fritsch R., Walther C. and Gruss P. (1996). Forebrain patterning defects in Small eye mutant mice. *Development* 122, 3453. 8951061 10.1242/dev.122.11.3453

[DMM045344C87] Sutovsky P. (2011). Sperm proteasome and fertilization. *Reproduction* 142, 1-14. 10.1530/REP-11-0041 21606061

[DMM045344C88] Swiderski R. E., Agassandian K., Ross J. L., Bugge K., Cassell M. D. and Yeaman C. (2012). Structural defects in cilia of the choroid plexus, subfornical organ and ventricular ependyma are associated with ventriculomegaly. *Fluids Barriers CNS* 9, 22-22. 10.1186/2045-8118-9-22 23046663 PMC3527152

[DMM045344C89] Ta-Shma A., Hjeij R., Perles Z., Dougherty G. W., Abu Zahira I., Letteboer S. J. F., Antony D., Darwish A., Mans D. A., Spittler S. et al. (2018). Homozygous loss-of-function mutations in MNS1 cause laterality defects and likely male infertility. *PLoS Genet.* 14, e1007602 10.1371/journal.pgen.1007602 30148830 PMC6128653

[DMM045344C90] Takeuchi F., Nabika T., Isono M., Katsuya T., Sugiyama T., Yamaguchi S., Kobayashi S., Yamori Y., Ogihara T. and Kato N. (2009). Evaluation of genetic loci influencing adult height in the Japanese population. *J. Hum. Genet.* 54, 749-752. 10.1038/jhg.2009.99 19834501

[DMM045344C91] Teves M. E., Zhang Z., Costanzo R. M., Henderson S. C., Corwin F. D., Zweit J., Sundaresan G., Subler M., Salloum F. N., Rubin B. K. et al. (2013). Sperm-associated antigen-17 gene is essential for motile cilia function and neonatal survival. *Am. J. Respir. Cell Mol. Biol.* 48, 765-772. 10.1165/rcmb.2012-0362OC 23418344 PMC3727877

[DMM045344C92] Teves M. E., Sundaresan G., Cohen D. J., Hyzy S. L., Kajan I., Maczis M., Zhang Z., Costanzo R. M., Zweit J., Schwartz Z. et al. (2015). Spag17 deficiency results in skeletal malformations and bone abnormalities. *PLoS ONE* 10, e0125936 10.1371/journal.pone.0125936 26017218 PMC4446355

[DMM045344C93] Town T., Breunig J. J., Sarkisian M. R., Spilianakis C., Ayoub A. E., Liu X., Ferrandino A. F., Gallagher A. R., Li M. O., Rakic P. et al. (2008). The stumpy gene is required for mammalian ciliogenesis. *Proc. Natl. Acad. Sci. USA* 105, 2853-2858. 10.1073/pnas.0712385105 18287022 PMC2268549

[DMM045344C94] Uechi H., Hamazaki J. and Murata S. (2014). Characterization of the testis-specific proteasome subunit α4s in mammals. *J. Biol. Chem.* 289, 12365-12374. 10.1074/jbc.M114.558866 24668818 PMC4007433

[DMM045344C95] van der Valk R. J. P., Kreiner-Møller E., Kooijman M. N., Guxens M., Stergiakouli E., Sääf A., Bradfield J. P., Geller F., Hayes M. G., Cousminer D. L. et al. (2014). A novel common variant in DCST2 is associated with length in early life and height in adulthood. *Hum. Mol. Genet.* 24, 1155-1168. 10.1093/hmg/ddu510 25281659 PMC4447786

[DMM045344C96] Vieira J. P., Lopes P. and Silva R. (2012). Primary ciliary dyskinesia and hydrocephalus with aqueductal stenosis. *J. Child Neurol.* 27, 938-941. 10.1177/0883073811429856 22290861

[DMM045344C97] Wallmeier J., Frank D., Shoemark A., Nöthe-Menchen T., Cindric S., Olbrich H., Loges N. T., Aprea I., Dougherty G. W., Pennekamp P. et al. (2019). De novo mutations in FOXJ1 result in a motile ciliopathy with hydrocephalus and randomization of left/right body asymmetry. *Am. J. Hum. Genet.* 105, 1030-1039. 10.1016/j.ajhg.2019.09.022 31630787 PMC6849114

[DMM045344C98] Wargo M. J., Dymek E. E. and Smith E. F. (2005). Calmodulin and PF6 are components of a complex that localizes to the C1 microtubule of the flagellar central apparatus. *J. Cell Sci.* 118, 4655 10.1242/jcs.02585 16188941

[DMM045344C99] Weedon M. N. and Frayling T. M. (2008). Reaching new heights: insights into the genetics of human stature. *Trends Genet.* 24, 595-603. 10.1016/j.tig.2008.09.006 18950892

[DMM045344C100] Weedon M. N., Lango H., Lindgren C. M., Wallace C., Evans D. M., Mangino M., Freathy R. M., Perry J. R. B., Stevens S., Hall A. S. et al. (2008). Genome-wide association analysis identifies 20 loci that influence adult height. *Nat. Genet.* 40, 575 10.1038/ng.121 18391952 PMC2681221

[DMM045344C101] Wessels M. W., den Hollander N. S. and Willems P. J. (2003). Mild fetal cerebral ventriculomegaly as a prenatal sonographic marker for Kartagener syndrome. *Prenat. Diagn.* 23, 239-242. 10.1002/pd.551 12627427

[DMM045344C102] Wirschell M., Nicastro D., Porter M. E. and Sale W. S. (2009). Chapter 9 - The regulation of axonemal bending. In *The Chlamydomonas Sourcebook*, 2nd edn. (ed. Harris E. H., Stern D. B. and Witman G. B.), pp. 253-282. London: Academic Press.

[DMM045344C103] Wood A. R., Esko T., Yang J., Vedantam S., Pers T. H., Gustafsson S., Chu A. Y., Estrada K., Luan J. A., Kutalik Z. et al. (2014). Defining the role of common variation in the genomic and biological architecture of adult human height. *Nat. Genet.* 46, 1173 10.1038/ng.3097 25282103 PMC4250049

[DMM045344C104] Xu X., Sha Y.-W., Mei L.-B., Ji Z.-Y., Qiu P.-P., Ji H., Li P., Wang T. and Li L. (2018). A familial study of twins with severe asthenozoospermia identified a homozygous SPAG17 mutation by whole-exome sequencing. *Clin. Genet.* 93, 345-349. 10.1111/cge.13059 28548327

[DMM045344C105] Yang P. and Smith E. F. (2009). Chapter 7 - The flagellar radial spokes. In *The Chlamydomonas Sourcebook*, 2nd edn. (ed. Harris E. H., Stern D. B. and Witman G. B.), pp. 209-234. London: Academic Press.

[DMM045344C106] Yang L., Banerjee S., Cao J., Bai X., Peng Z., Chen H., Huang H., Han P., Feng S., Yi N. et al. (2018). Compound heterozygous variants in the coiled-coil domain containing 40 gene in a chinese family with primary ciliary dyskinesia cause extreme phenotypic diversity in cilia ultrastructure. *Front. Genet.* 9 10.3389/fgene.2018.00023 PMC5801289 29456554

[DMM045344C107] Zhang Z., Jones B. H., Tang W., Moss S. B., Wei Z., Ho C., Pollack M., Horowitz E., Bennett J., Baker M. E. et al. (2005). Dissecting the Axoneme Interactome. *Mol. Cell. Proteomics* 4, 914 10.1074/mcp.M400177-MCP200 15827353

[DMM045344C108] Zhang Z., Zariwala M. A., Mahadevan M. M., Caballero-Campo P., Shen X., Escudier E., Duriez B., Bridoux A.-M., Leigh M., Gerton G. L. et al. (2007). A heterozygous mutation disrupting the SPAG16 gene results in biochemical instability of central apparatus components of the human sperm axoneme. *Biol. Reprod.* 77, 864-871. 10.1095/biolreprod.107.063206 17699735

[DMM045344C109] Zhang S., Jiang E., Wang K., Zhang Y., Yan H., Qu L., Chen H., Lan X. and Pan C. (2019). Two Insertion/deletion variants within SPAG17 gene are associated with goat body measurement traits. *Animals (Basel)* 9, 379 10.3390/ani9060379 PMC6616450 31234269

[DMM045344C110] Zhao J., Li M., Bradfield J. P., Zhang H., Mentch F. D., Wang K., Sleiman P. M., Kim C. E., Glessner J. T., Hou C. et al. (2010). The role of height-associated loci identified in genome wide association studies in the determination of pediatric stature. *BMC Med. Genet.* 11, 96 10.1186/1471-2350-11-96 20546612 PMC2894790

[DMM045344C111] Zhao L., Hou Y., Picariello T., Craige B. and Witman G. B. (2019). Proteome of the central apparatus of a ciliary axoneme. *J. Cell Biol.* 218, 2051-2070. 10.1083/jcb.201902017 31092556 PMC6548120

[DMM045344C112] Zhu X., Poghosyan E., Rezabkova L., Mehall B., Sakakibara H., Hirono M., Kamiya R., Ishikawa T. and Yang P. (2019). The roles of a flagellar HSP40 ensuring rhythmic beating. *Mol. Biol. Cell* 30, 228-241. 10.1091/mbc.E18-01-0047 30427757 PMC6589562

[DMM045344C113] Zimmerman S. and Sutovsky P. (2009). The sperm proteasome during sperm capacitation and fertilization. *J. Reprod. Immunol.* 83, 19-25. 10.1016/j.jri.2009.07.006 19853307

