# Correction: A novel hypomorphic allele of *Spag17* causes primary ciliary dyskinesia phenotypes in mice

**DOI:** 10.1242/dmm.048645

**Published:** 2021-01-11

**Authors:** Zakia Abdelhamed, Marshall Lukacs, Sandra Cindric, Saima Ali, Heymut Omran, Rolf W. Stottmann

**Affiliations:** 1 Division of Human Genetics, Cincinnati Children's Hospital Medical Center, Cincinnati, OH 45229, USA; 2 Department of Anatomy and Embryology, Faculty of Medicine (Girl's Section), Al-Azhar University, Cairo 11651, Egypt; 3 Medical Scientist Training Program, Cincinnati Children's Hospital Medical Center, Cincinnati, OH 45229, USA; 4 Department of General Pediatrics, University Children's Hospital Münster, 48149 Münster, Germany; 5 Division of Developmental Biology, Cincinnati Children's Hospital Medical Center, Cincinnati, OH 45229, USA; 6 Department of Pediatrics, University of Cincinnati, Cincinnati, OH 45229, USA

There was an error in Dis. Model. Mech. (2020) 13, dmm045344 (doi:10.1242/dmm.045344).

**Author contributions**

Conceptualization: Z.A., R.W.S.; Formal analysis: Z.A., R.W.S., S.C.; Investigation: Z.A., M.L., S.A., H.O., S.C.; Resources: R.W.S.; Data curation: Z.A., S.A., R.W.S.; Writing - original draft: Z.A., R.W.S., M.L., S.C.; Writing - review & editing: Z.A., R.W.S., H.O.; Supervision: R.W.S., H.O.; Project administration: R.W.S.; Funding acquisition: R.W.S., H.O.

Saima Ali was inadvertently left off the list of authors for this paper. The complete list of authors, their affiliations and contributions are shown above.

The corresponding author would like to apologise for this error. Both the online full text and PDF versions of the paper have been corrected.

