# Supplementary material for: A novel hypomorphic allele of Spag17 causes primary ciliary dyskinesia phenotypes in mice
Source: Dis Model Mech. 2020 Oct 30;13(10):dmm045344. doi: 10.1242/dmm.045344 (PMC7648611; doi:10.1242/dmm.045344)
Supplement: Supplementary information [file dmm-13-045344-s1.pdf]

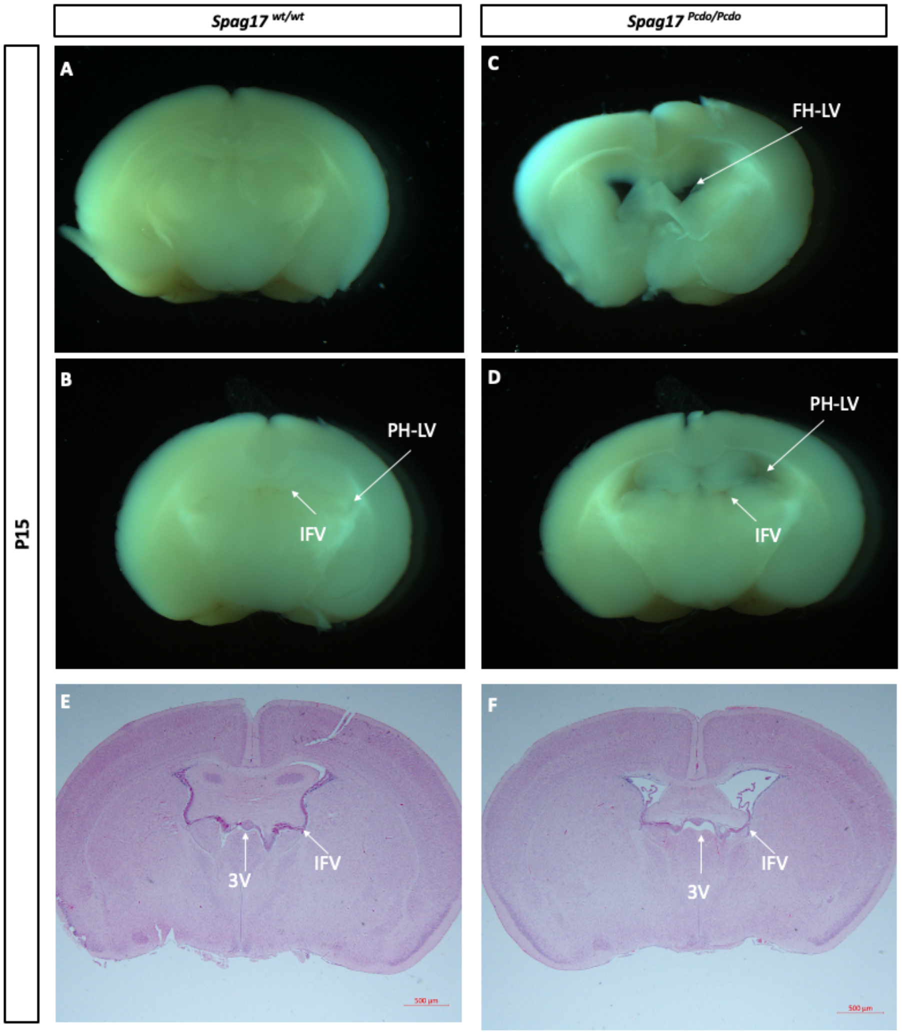

Figure S1. No masses or hemorrhage are involved in the development of the hydrocephalus phenotype in the *Pcdo* mutants.

Whole mount brain slices (A-D) from *Spag17*$^{wt/wt}$ (A,B, n=5) and *Spag17* $^{Pcdo/Pcdo}$ (C,D, n=6) P15 animals. Pictures indicate dilation of the lateral ventricle in mutants, with no abnormal masses or blood clots visible in either the frontal horn (FH-LV) or the posterior horn of the lateral ventricle (PH-LV), or the interventricular foramen (IVF). (E,F) Histological preparations confirm these observations from the whole mount analysis and additionally show the enlargement of the third ventricle (3V) at P15 in the *Spag17* $^{Pcdo/Pcdo}$ (F, n=6) animals as compared to *Spag17*$^{wt/wt}$ (E, n=8). Scale bar= 500µm in E and F.

Disease Models & Mechanisms • Supplementary information

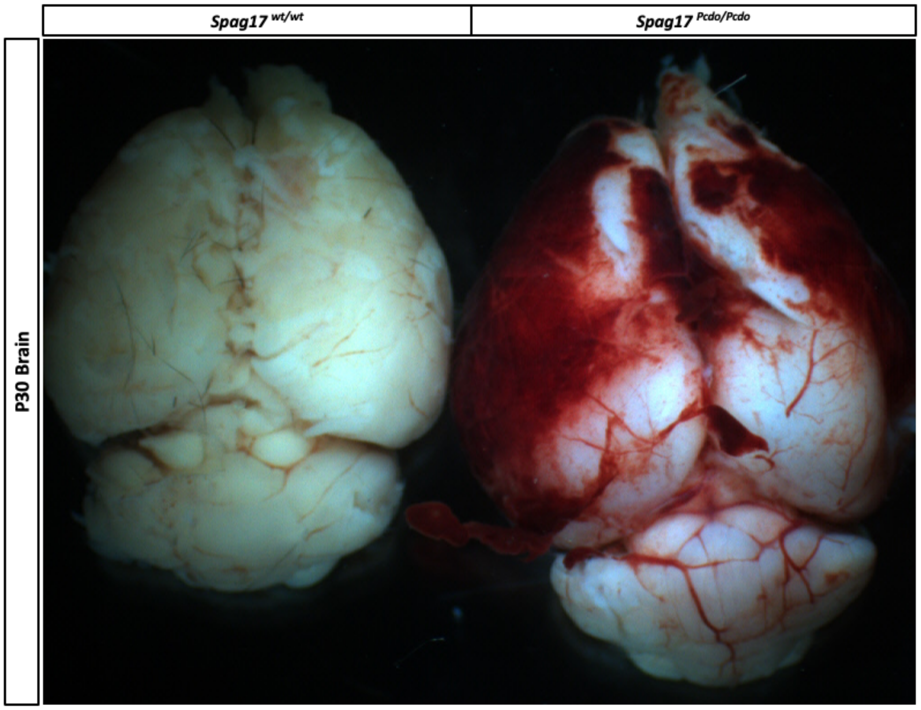

Figure S2. Intraventricular hemorrhage (IVH) was observed in less than 10% of the Spag17 $^{Pcdo/Pcdo}$ mutant animals.

Whole brain image of wildtype and *Spag17*$^{Pcdo/pcdo}$ mutants that incidentally developed severe obvious IVH. This is noted in animals at one month of age and older, n=3/32 mutants.

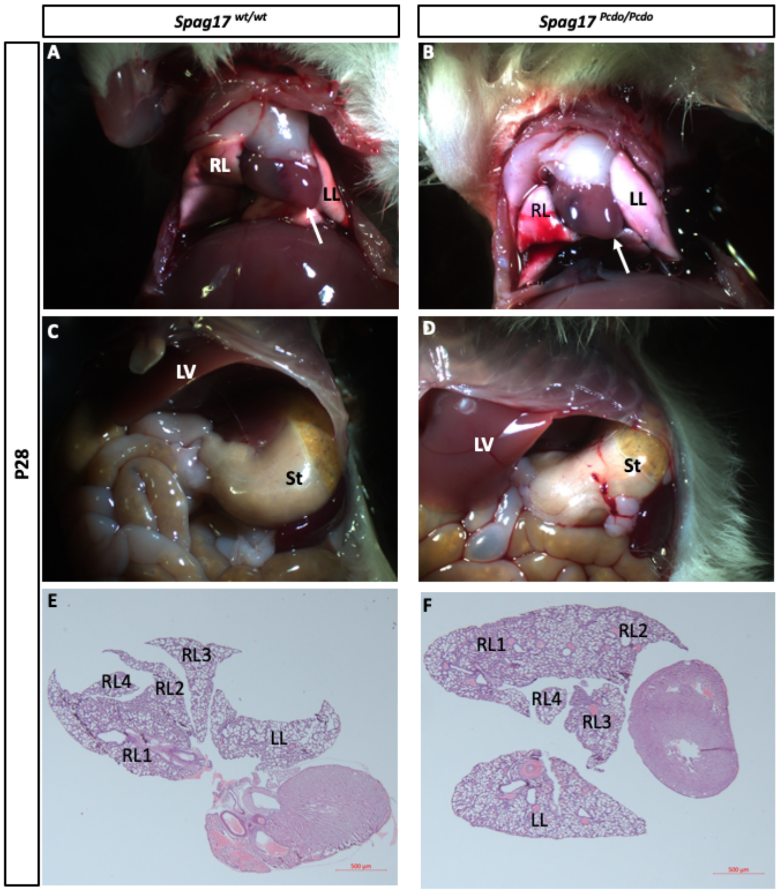

Figure S3. No organ laterality defects nor lung isomerism in the *Spag17*^{Pcdo} mutants.

Pictures of the exposed thoracic and abdominal cavity from *Spag17*$^{wt/wt}$ (A,C, n=5) and *Spag17* $^{Pcdo/Pcdo}$ (B,D, n=5), showing the normal positioning of the heart apex (white arrow) to the left side of the thoracic cavity close to the left lung (LL), normal position of the stomach (St), normal position of the liver (Lv), and right lung (RL). (E,F) Histology of cross sections through the thoracic cavity of P1 *Spag17*$^{wt/wt}$ (E, n=18) and *Spag17* $^{Pcdo/Pcdo}$ (F, n=15), showing no right or left lung isomerism with the right lung formed of 4 lobes (RL1-RL4) and the left lung formed of one lung lobe (LL). Scale bar= 500µm in (G and H).

Disease Models & Mechanisms • Supplementary information

Table S1. Exome sequencing of *Pcdo* mutants.

	mut 1	mut 2	mut 3
total variants on exome	118,893	126,146	114,194
homozygous	52,734	73,800	56,014
not in db SNP (i.e., not recorded strain polymorphism)	8,665	12,779	9,874
predicted high impact	10	14	7
shared among all three mutants	3 genes: *Plxnc1, Sfi1, Spag17*		

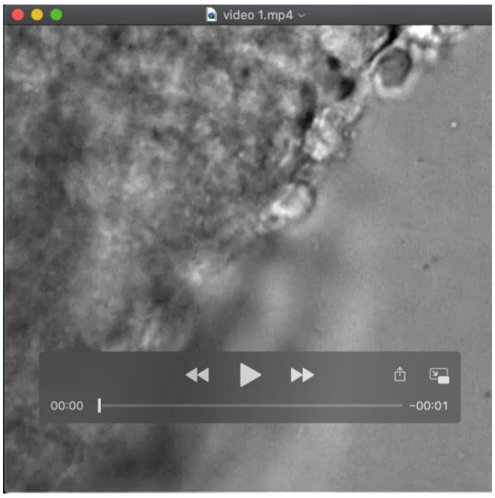

Movie 1. Video microscopy analysis of the wild-type forebrain medial wall of ependymal cilia. High speed video recording of the P4 *Spag17*$^{wt/wt}$ ependymal cilia of the medial wall of the lateral ventricle. 300 frames acquired at a rate of 400 per second

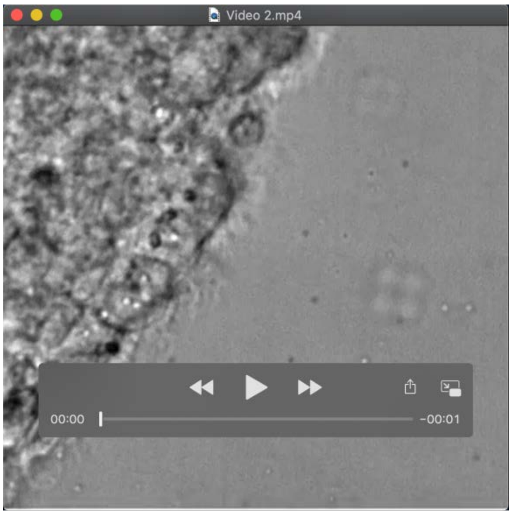

Movie 2. Video microscopy analysis of the *Spag17*$^{Pcdo/Pcdo}$ forebrain medial wall of ependymal cilia. High speed video recording of the p4 *Spag17*$^{Pcdo/Pcdo}$ ependymal cilia of the medial wall of the lateral ventricle. 300 frames acquired at a rate of 400 frames per second.

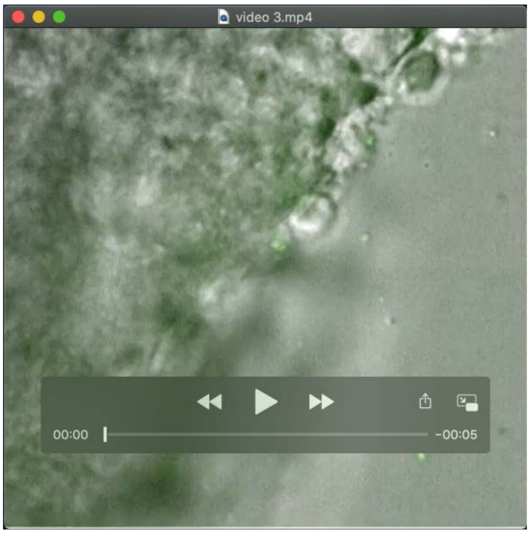

Movie 3. Green fluorescent microbeads flow traced in the wild-type forebrain medial wall. Green fluorescent light alternating with DIC white light to generate high speed video microscopy recording to show ependymal cilia beating simultaneously with the moving green flourcent beads and the beating cilia along the medial wall of the P4 medial wall of the lateral ventricle of the *Spag17*$^{wt/wt}$ animals. 300 frames captured at a rate of 66 frames per second

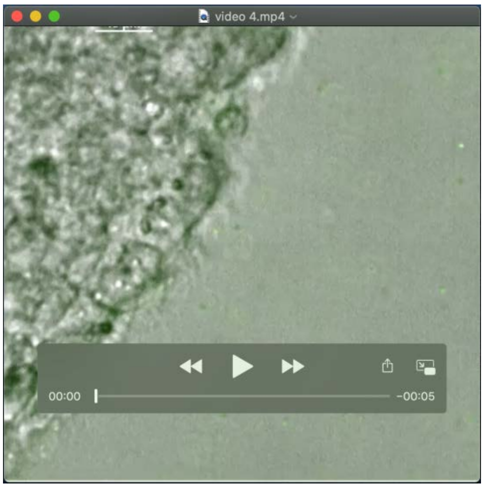

Movie 4. Green fluorescent microbeads flow recording in the *Spag17*$^{Pcdo/Pcdo}$ forebrain medial wall. Green fluorescent light alternating with DIC white light to generate high speed video microscopy recording ependymal cilia beating simultaneously with the moving green flourcent beads and the beating cilia along the medial wall of the P4 medial wall of the lateral ventricle of the *Spag17*$^{Pcdo/Pcdo}$ mutant animals. 300 frames captured at a rate of 66 frames per second

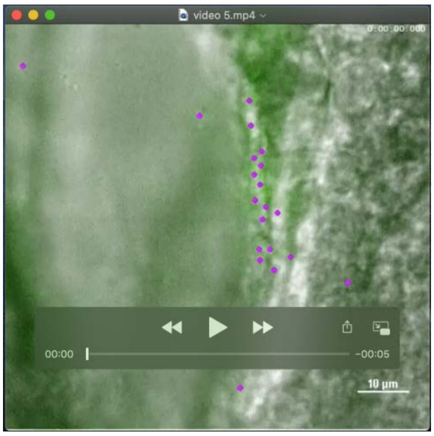

Movie 5. Green fluorescent microbeads flow traced in the wild-type aqueduct of Sylvius. Green fluorescent light alternating with DIC white light to generate high speed video microscopy recording showing ependymal cilia beating simultaneously with the moving green flourcent beads and the beating cilia of the intact aqueduct slice of wild type animals. 300 frames captured at a rate of 66 frames per second

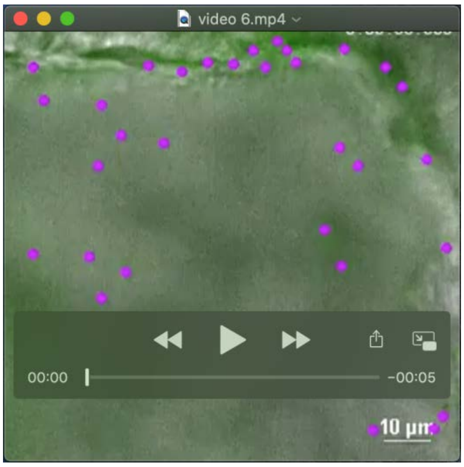

Movie 6. Green fluorescent microbeads flow recording in the *Spag17*$^{Pcdo/Pcdo}$ aqueduct of Sylvius. Green fluorescent light alternating with DIC white light to generate high speed video microscopy recording showing ependymal cilia beating simultaneously with the moving green flourcent beads and the beating cilia of the intact aqueduct slice of *Spag17*$^{Pcdo/Pcdo}$ mutant animals. 300 frames captured at a rate of 66 frames per second. The flowing beads seem to move in circles with reduced flow speed.

Disease Models & Mechanisms • Supplementary information
